# *PTCH1*-mutant human cerebellar organoids exhibit altered neural development and recapitulate early medulloblastoma tumorigenesis

**DOI:** 10.1242/dmm.050323

**Published:** 2024-02-27

**Authors:** Max J. van Essen, Elizabeth J. Apsley, Joey Riepsaame, Ruijie Xu, Paul A. Northcott, Sally A. Cowley, John Jacob, Esther B. E. Becker

**Affiliations:** ^1^ Nuffield Department of Clinical Neurosciences, University of Oxford, Oxford OX3 9DU, UK; ^2^ Kavli Institute of Nanoscience Discovery, University of Oxford, Oxford OX1 3QU, UK; ^3^ Genome Engineering Oxford, Sir William Dunn School of Pathology, University of Oxford, South Parks Road, OX1 3RE Oxford, UK; ^4^ Department of Developmental Neurobiology, St. Jude Children's Research Hospital, 262 Danny Thomas Place, Memphis, TN 38105-3678, USA; ^5^ James and Lillian Martin Centre for Stem Cell Research, Sir William Dunn School of Pathology, University of Oxford, South Parks Road, OX1 3RE, UK

**Keywords:** Cerebellum, Development, Patched 1, Sonic hedgehog, Medulloblastoma, iPSCs, CRISPR

## Abstract

Patched 1 (PTCH1) is the primary receptor for the sonic hedgehog (SHH) ligand and negatively regulates SHH signalling, an essential pathway in human embryogenesis. Loss-of-function mutations in *PTCH1* are associated with altered neuronal development and the malignant brain tumour medulloblastoma. As a result of differences between murine and human development, molecular and cellular perturbations that arise from human *PTCH1* mutations remain poorly understood. Here, we used cerebellar organoids differentiated from human induced pluripotent stem cells combined with CRISPR/Cas9 gene editing to investigate the earliest molecular and cellular consequences of *PTCH1* mutations on human cerebellar development. Our findings demonstrate that developmental mechanisms in cerebellar organoids reflect *in vivo* processes of regionalisation and SHH signalling, and offer new insights into early pathophysiological events of medulloblastoma tumorigenesis without the use of animal models.

## Teaser

**Summary:** Cerebellar organoids recapitulate *in vivo* processes of regionalisation and SHH signalling, and offer new insight into early pathophysiological events of medulloblastoma tumorigenesis without the use of animal models.

## INTRODUCTION

Patched 1 (PTCH1) is the primary receptor for the sonic hedgehog (SHH) ligand and is the most proximal negative regulator of the SHH signal transduction pathway. SHH signalling is essential in human embryogenesis and regulates differentiation and proliferation of target cells (Fuccillo et al., 2006). During cerebellar development, SHH activity regulates the proliferative expansion of cerebellar granule cell progenitors (GCPs) in the external granular layer (EGL) of the cerebellum (Aguilar et al., 2012; Dahmane and Ruiz i Altaba, 1999; Leto et al., 2016; Wechsler-Reya and Scott, 1999). PTCH1 plays a pivotal role in controlling SHH-induced proliferation by preventing smoothened (SMO) from transducing SHH signals (Blassberg and Jacob, 2017; Corbit et al., 2005; Corcoran and Scott, 2006; Denef et al., 2000; Dwyer et al., 2007; Khaliullina et al., 2009; Nachtergaele et al., 2012). Consequently, PTCH1 loss of function (LOF) leads to constitutive SMO activation and SHH signalling. SHH signalling primarily occurs through post-transcriptional modulation of GLI proteins, a class of transcription factors that regulate stem cell maintenance, proliferation and differentiation (Ruiz i Altaba et al., 2002a).

*PTCH1* LOF gene mutations are associated with various types of cancer (Ruiz i Altaba et al., 2002b), including the cerebellar brain tumour medulloblastoma (MB), the most frequent malignant brain tumour in children (Ostrom et al., 2018; Smoll and Drummond, 2012). A major MB subtype arises from overactive SHH signalling in GCPs, classified as SHH-MB. Notably, around 44% of all SHH-MB tumours harbour heterozygous LOF mutations in *PTCH1* (Garcia-Lopez et al., 2021; Northcott et al., 2019). Mouse and human studies have helped to identify GCPs as the cells of origin of SHH-MB (Hovestadt et al., 2019; Vladoiu et al., 2019; Yang et al., 2008), but early molecular and cellular perturbations resulting from human *PTCH1* mutations that drive later malignant transformation remain incompletely understood. Increasing evidence reveals how genetic events have different effects in mice compared to those in humans, emphasising the importance of developing tools to study human development and disease (Bouaoun et al., 2016; Jacks et al., 1994). Furthermore, studies in recent years have shed light on relevant differences between murine and human cerebellar and MB development. Specifically, Purkinje cells in mice secrete Shh that acts on GCPs to induce proliferation (Dahmane and Ruiz i Altaba, 1999), whereas human Purkinje cells are likely not mature enough to produce SHH when SHH-induced proliferation first occurs (Zecevic and Rakic, 1976). Thus, human-specific models are needed to better understand PTCH1-mediated signalling relevant to human disease and to make further advances in MB treatment.

Induced pluripotent stem cell (iPSC) technology has transformed the way human development and disease is studied and opened new human-centric avenues for disease modelling. Unlike transformed cell lines, iPSCs allow *in vitro* study of human cellular differentiation and physiology under conditions of a normal karyotype, gene dosage, cell cycle and metabolic profile. Among the numerous cell types and tissues that can now be generated from iPSCs *in vitro* are cerebellar neurons and organoids that recapitulate early hindbrain patterning and cerebellar lineage formation (Muguruma et al., 2015; Watson et al., 2018). Single-cell RNA sequencing of human iPSC-derived, xeno-free cerebellar organoids maintained for 90 days *in vitro* revealed the presence of all major cerebellar developmental neuronal cell types, including rhombic lip (RL) cells, GCPs, and proliferating and post-mitotic granule cells (GCs) (Nayler et al., 2021). Here, we aimed to investigate the earliest molecular and cellular consequences of *PTCH1* mutations on human cerebellar development and without the use of animal models by combining cerebellar organoid technology with CRISPR/Cas9 gene editing.

CRISPR gene editing has emerged as an exciting tool for functional gene studies and disease modelling (van Essen et al., 2021). Using CRISPR technology, mutant iPSC lines can be generated and compared to isogenic healthy controls, which avoids the confounding effect of different genetic backgrounds on the mutant cellular phenotype. Although groups have modelled SHH-MB using neuroepithelial stem cells differentiated from iPSCs from patients carrying *PTCH1* mutations (Huang et al., 2019; Susanto et al., 2020), these studies required orthotopic injection in mice. Others have modelled high-grade glioma and MB in forebrain and cerebellar organoids by overexpressing driver genes through electroporation (Bian et al., 2018; Lago et al., 2023; Ogawa et al., 2018). However, this method favours highly aggressive and late-stage genotypes and does not recapitulate germline tumour predisposition. Our approach of directing *PTCH1-*heterozygous and -homozygous mutant iPSCs to cerebellar organoid differentiation allows the investigation of the effects of *PTCH1* dysfunction on cerebellar development for the first time in a human model *in vitro*, without influences of the murine host environment (Ben-David et al., 2017). We found that homozygous *PTCH1* LOF in iPSCs prevented cerebellar differentiation and promoted ventral forebrain identity through early high-level SHH signalling. In contrast, *PTCH1-*heterozygous iPSCs differentiated into cerebellar organoids that harboured an expanded RL and GCP population and displayed features associated with pre-neoplastic stages of MB. Taken together, these results illustrate the utility of cerebellar organoids in studying the effects of human gene mutations on development and disease.

## RESULTS

### Introduction of a *PTCH1* LOF mutation in human iPSCs

**Fig. 1.**
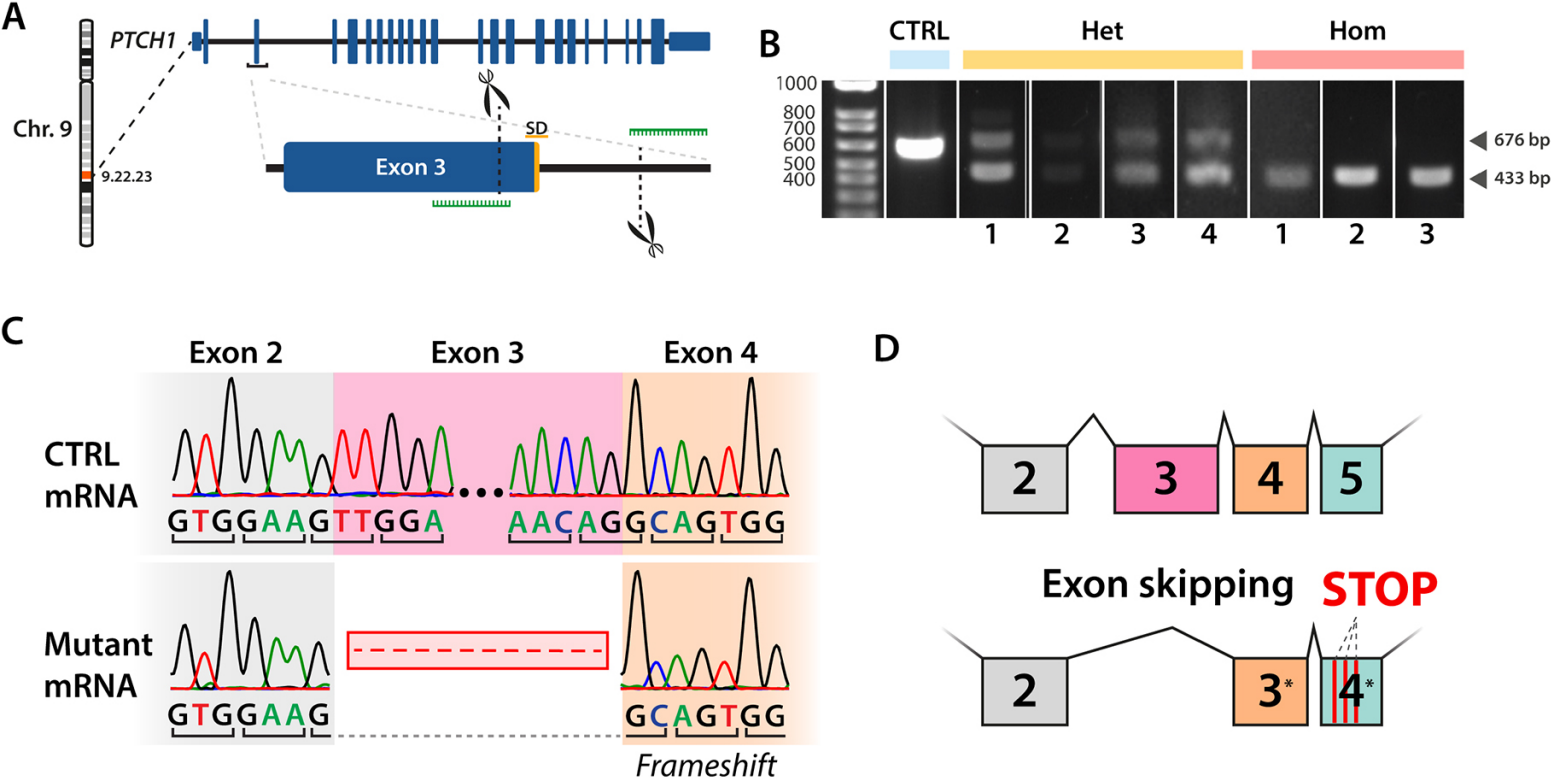
**Generation of *PTCH1* loss-of-function mutations in human iPSCs using CRISPR.** (A) CRISPR gene editing strategy to target exon 3 of the *PTCH1* gene. Guide RNAs (green) were designed to result in the excision of a 243-bp region spanning the splice donor (SD) site of exon 3. (B) PCR genotyping confirmed the generation of heterozygous (Het) *PTCH1* iPSC clones producing both control (CTRL) (676 bp) and mutant (433 bp) PCR amplicons as well as homozygous (Hom) clones producing only the mutant amplicon. (C) Sanger sequencing of *PTCH1* mRNA revealed the exclusion of exon 3 in the mutant mRNA, resulting in a frameshift mutation in exon 4. (D) Schematic depiction of the effect of exon 3 exclusion, resulting in a frameshift mutation and multiple premature translation termination codons in the new exon 4. See also Figs S1-S6.

To generate *PTCH1-*mutant human iPSCs, we targeted exon 3 of *PTCH1* (a gene with 24 exons), which is retained in all main protein-coding splice isoforms and encodes part of the first extracellular loop that is thought to be an essential region of the protein. Using a pair of guide RNAs (gRNAs) and a Cas9 ribonucleoprotein approach, we excised the splice donor of this exon and generated *PTCH1-*mutant iPSCs (Fig. 1A). We established monoclonal heterozygous and homozygous iPSC lines by single-cell cloning on mouse embryonic feeder cells. Each line was genotyped using PCR to detect cutting at the gRNA target sites (Fig. 1B), which was confirmed by Sanger sequencing (Fig. S1). Examination of cDNA generated through reverse transcription of *PTCH1* mRNA from mutant iPSC clones revealed that CRISPR/Cas9 induced alternative splicing and exclusion of exon 3 (Fig. 1C,D). As exon 3 is an asymmetric exon (not a multiple of three base pairs), this resulted in a −1 frameshift in the mutant mRNA with multiple premature stop-codons in the new reading frame and thus a *PTCH1*-null mutant. The underlying mechanism for this is likely protein truncation rather than nonsense-mediated decay as elevated levels of *PTCH1* mRNA were present in the mutant iPSC clones (Fig. S6A).

*PTCH1-*mutant clones maintained iPSC morphology and expressed NANOG and the Tra-1-60 antigen (PODXL) to similar levels, as measured by flow cytometry (Fig. S2). A screen for chromosomal aberrations using a single nucleotide polymorphism (SNP) array testing over 600,000 sites in the human genome did not reveal any new amplifications, deletions or re-arrangements (Fig. S3). Furthermore, no specific off-target effects of the CRISPR strategy were detected upon sequencing of the top five off-target sites for each of the gRNAs (Fig. S4). iPSCs of all three genotypes could be differentiated into ectodermal, endodermal and mesodermal stem cells using directed differentiation (Fig. S5), confirming their pluripotency.

PTCH1 LOF is known to increase SHH signalling in SHH-responsive cells (Chen et al., 2002; Goodrich et al., 1997; Taipale et al., 2000). Although iPSCs are not known to be SHH responsive, we used reverse-transcription quantitative PCR (RT-qPCR) to measure mRNA levels of well-established readouts of SHH pathway activity, *GLI1* and *PTCH1* (Goodrich et al., 1996; Lee et al., 1997). GLI1 is one of the main effector proteins of the SHH pathway and functions as a transcription activator, whereas *PTCH1* gene expression is upregulated upon SHH pathway activation to serve as a negative feedback mechanism (Hui and Angers, 2011; Ingham et al., 2011). Both targets were modestly upregulated in homozygous (*PTCH1*^−/−^) iPSCs compared to their expression in isogenic control cells (*GLI1*, *P*<0.001, 95% c.i. [−2.9498, −1.0680]; *PTCH1*, *P*=0.01, 95% c.i. [−1.87930, −0.23886]; Fig. S6A), consistent with the absence of PTCH1–SHH signalling. In heterozygous (*PTCH1*^+/−^) iPSCs, only *GLI1* expression was elevated in comparison with that in control iPSCs (*P*=0.027, 95% c.i. [−2.0990, −0.2172]). To test whether these upregulated readouts of SHH signalling implied SHH induced proliferation of our iPSC lines, we analysed the proliferation of *PTCH1*-mutant iPSCs by exposing cells to a pulse of 5-ethynyl-2′-deoxyuridine (EdU). No statistical differences could be observed in the proportions of cells in different stages of the cell cycle (Fig. S6B). Taken together, these results show that *PTCH1-*mutant iPSCs maintain normal morphology, pluripotency and proliferation.

### Cerebellar differentiation of *PTCH1*-mutant iPSCs reveals changes in morphology and cerebellar gene expression

**Fig. 2.**
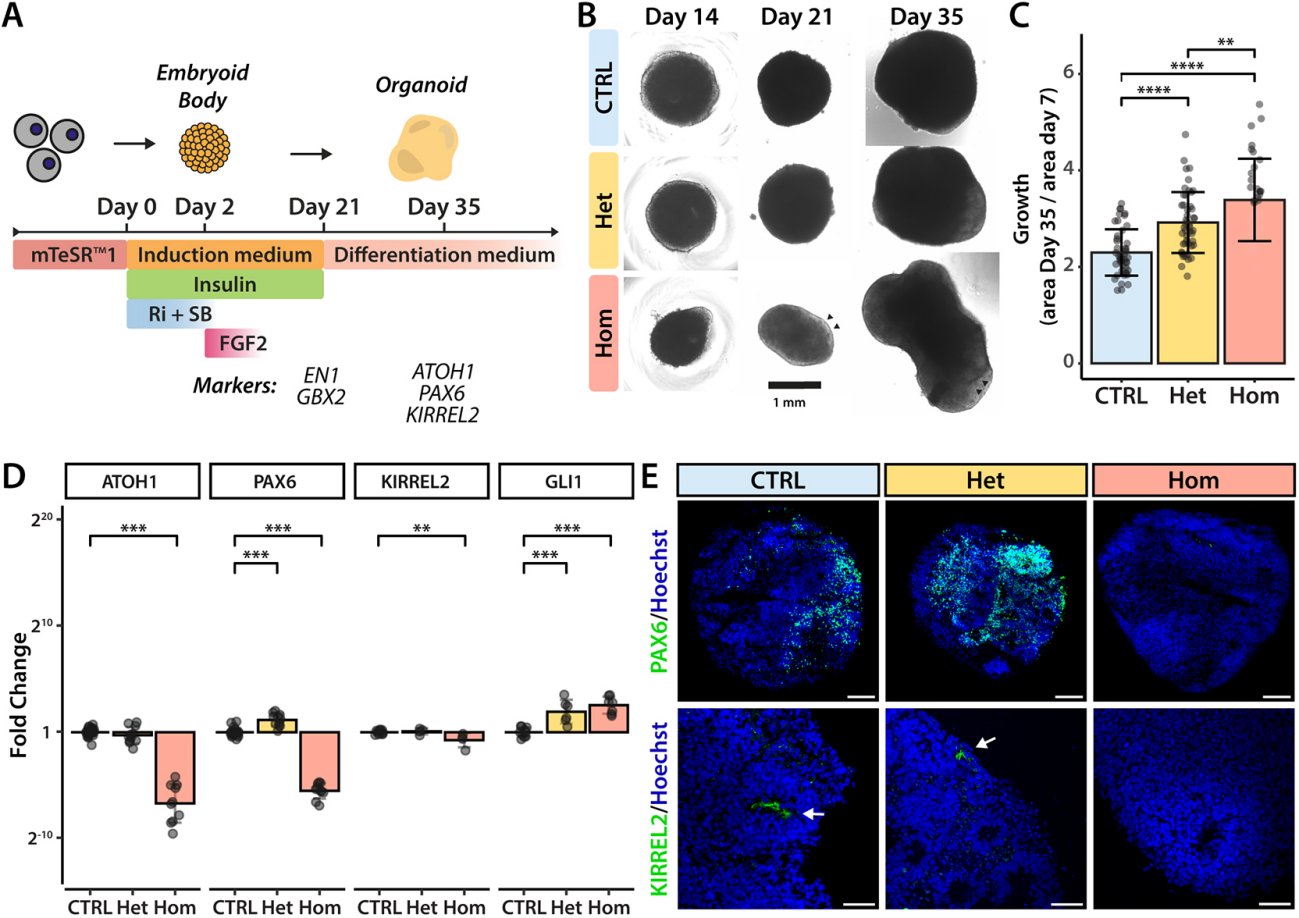
**Cerebellar differentiation of *PTCH1*-mutant iPSCs reveals changes in morphology and cerebellar lineage markers.** (A) Schematic overview of the cerebellar differentiation protocol. Ri, Rho kinase inhibitor Y-27632; SB, SB431542. (B) Representative images of organoids generated from control (CTRL), *PTCH1^+/−^* (Het) and *PTCH1^−/−^* (Hom) iPSCs during differentiation. Arrowheads indicate regions of enhanced polarisation within the homozygous organoids. (C) Relative size change of organoids at day 35 compared to day 7. The data are from 46 (CTRL), 48 (Het) and 35 (Hom) individual organoids across three differentiations. Error bars indicate s.d. Statistical significance was computed by one-way ANOVA [*F*(2,126)=28.31, *P*<0.001] with Tukey's post hoc test (CTRL versus Het, *P*<0.001, 95% c.i. [0.3011, 0.9412]; CTRL versus Hom, *P*<0.001, 95% c.i. [0.7406, 1.4364]; Het versus Hom, *P*=0.005, 95% c.i. [0.1226, 0.8121]). (D) Expression of cerebellar and SHH pathway genes, measured by RT-qPCR. Each data point represents an experimental replicate. Data are from at least three biological replicates from separate differentiations. Error bars indicate s.d. Statistical significance was computed by one-way ANOVA: *ATOH1* [*F*(2,46)=227.272, *P*<0.001], Dunnett's post hoc test (CTRL versus Hom, *P*<0.001, 95% c.i. [5.9792, 7.4879]); *PAX6* [*F*(2,45)=581.687, *P*<0.001], Dunnett's post hoc test (CTRL versus Het, *P*<0.001, 95% c.i. [−1.5630, −0.6926]; CTRL versus Hom, *P*<0.001, 95% c.i. [5.0842, 5.9291]); *KIRREL2* [*F*(2,19)=6.141, *P*=0.009], Dunnett's post hoc test (CTRL versus Hom, *P*=0.009, 95% c.i. [0.13853, 0.95927]); *GLI1* [*F*(2,20)=25.272, *P*<0.001], Dunnett's post hoc test (CTRL versus Het, *P*<0.001, 95% c.i. [−2.8026, −0.9513]; CTRL versus Hom, *P*<0.001, 95% c.i. [−3.3523, −1.5857]). **P<0.01; ***P<0.001; ****P<0.0001. (E) Immunofluorescence staining of day 35 organoids with antibodies specific to the rhombic lip lineage marker PAX6 (green) and the ventricular zone marker KIRREL2 (green). KIRREL2 is primarily expressed in the small neuroepithelial lumina present in cerebellar organoids (arrows). Nuclei are visualised in blue by Hoechst staining. Images are representative of six independent experiments. Scale bars: 100 μm (top); 50 μm (bottom). See also Fig. S2.

To investigate the effect of PTCH1 LOF on cerebellar differentiation, control and *PTCH1*-mutant human iPSC lines were differentiated into cerebellar organoids following a previously published protocol by our laboratory (Nayler et al., 2021) (Fig. 2A). Heterozygous organoids exhibited a similar shape to that of control organoids (Fig. 2B). In contrast, homozygous organoids showed changed morphology with more irregular growth and polarisation of the tissue, marked by greater translucency of the edges by brightfield imaging. Comparing the sizes of mutant versus control organoids, respectively, we found that *PTCH1*-heterozygous and -homozygous mutants grew more quickly compared to control organoids (Fig. 2C). These results are in line with previous studies that show neural overgrowth in *Ptch1*^+/−^ mice (Jackson et al., 2020; Oliver et al., 2005). The marked altered morphology of *PTCH1*^−/−^ organoids suggested changes in the differentiation trajectory of the homozygous mutant iPSCs.

We next assessed differentiation along the midbrain–hindbrain lineage by measuring mRNA levels of engrailed 1 (*EN1*), which marks cells with midbrain and anterior hindbrain region identity, and gastrulation brain homeobox 2 (*GBX2*), which is expressed by anterior hindbrain cells (Liu and Joyner, 2001). Expression of orthodenticle homeobox 2 (*OTX2*), which is expressed in the developing midbrain but is absent in the presumptive cerebellar territory, was also used to distinguish the two regions (Vernay et al., 2005). At day 21 of the differentiation protocol, the expression of *EN1* and *GBX2* was increased in organoids of all three genotypes compared to their expression levels in iPSCs, whereas *OTX2* levels remained low. Between the organoids of the different genotypes, only the expression of *EN1* was significantly higher in *PTCH1*^+/*−*^ (‘Het’) and *PTCH1*^−/−^ (‘Hom’) organoids compared to that in control (‘CTRL’) organoids (CTRL versus Het, *P*<0.001, 95% c.i. [−4.7184, −1.6742]; CTRL versus Hom, *P*<0.001, 95% c.i. [−4.7728, −1.7287]) (Fig. S7). To further explore the impact of *PTCH1* LOF on cerebellar induction, we analysed the expression of cerebellar lineage markers after 35 days of differentiation. Gene expression of *ATOH1* and *PAX6*, which mark the glutamatergic lineage, and *KIRREL2*, expressed by presumptive GABAergic neurons, was measured by RT-qPCR (Fig. 2D). Interestingly, we found that expression of both glutamatergic and GABAergic cerebellar markers was significantly lower in homozygous organoids compared to that in controls. Loss of PAX6 and KIRREL2 expression in homozygous organoids was confirmed by immunofluorescence (Fig. 2E). In contrast, heterozygous organoids expressed *ATOH1* and *KIRREL2* mRNA at similar levels to those in controls. In fact, the expression of *PAX6* mRNA was higher in heterozygous organoids, compared to that in control organoids (*P*<0.001), opposite to the effect seen in homozygous organoids. These changes were accompanied by increased SHH pathway activity in both heterozygous and homozygous genotypes as measured by the upregulated expression of *GLI1* (Fig. 2D). The lower expression of cerebellar lineage markers in *PTCH1*^−/−^ organoids suggested that cell patterning was severely disrupted. By contrast, the maintained expression of cerebellar markers in *PTCH1*^+/−^ heterozygous organoids implied that sufficient PTCH1 activity was present in these organoids to facilitate the initial stages of cerebellar development.

### Homozygous *PTCH1* LOF prevents cerebellar organoid differentiation

**Fig. 3.**
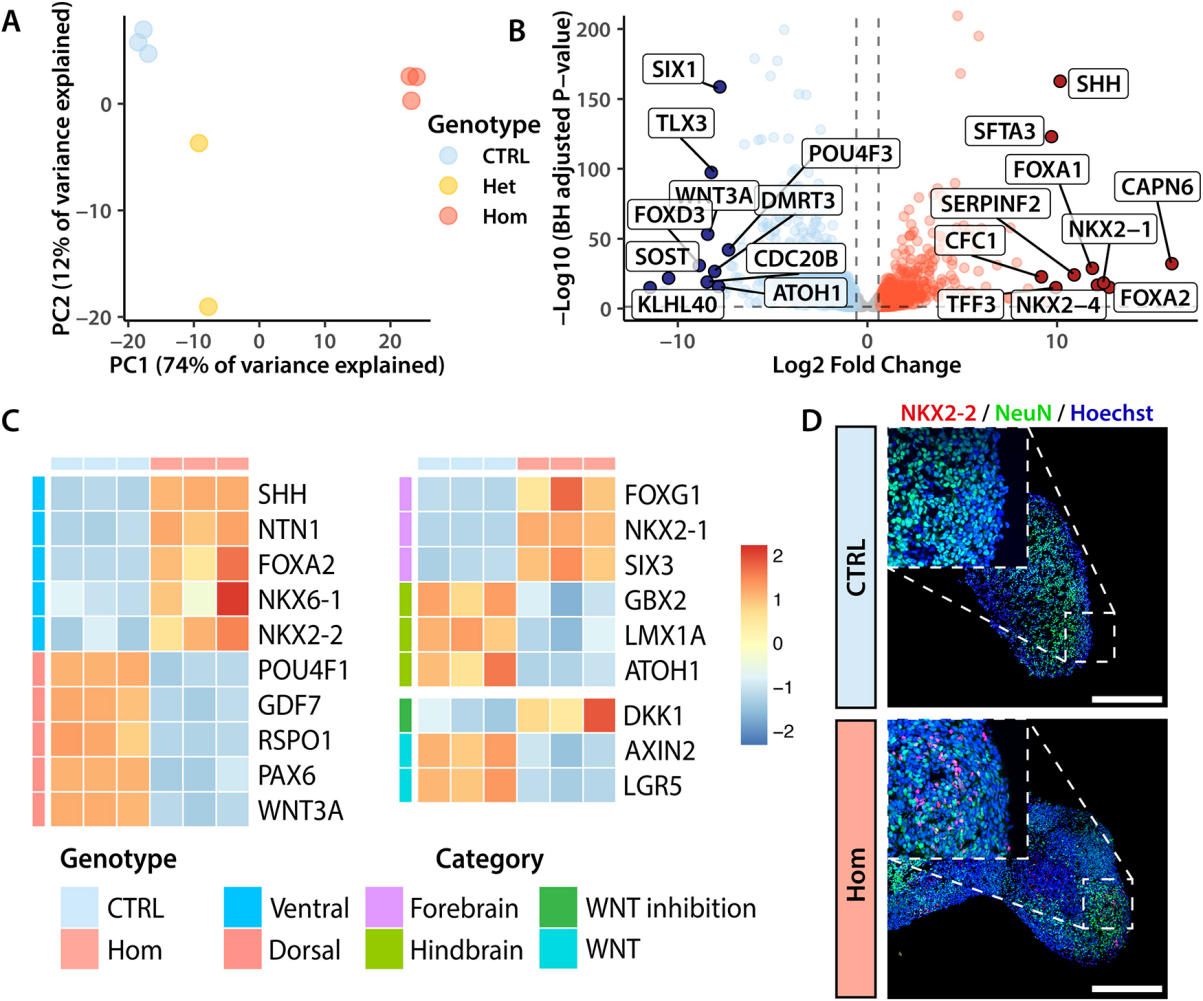
**Homozygous *PTCH1* loss-of-function prevents cerebellar organoid differentiation.** (A) Principal component analysis using the 1000 most variable genes showing the separation of samples in principal component (PC) 1 and PC2. Control (CTRL) samples were generated with the AH017 line, *PTCH1*^+/−^ (Het) samples with lines A3 and C6, and *PTCH1^−/−^* (Hom) samples with lines A2, B3 and H3. (B) Volcano plot of the ten genes with the most significant change in either direction. Genes upregulated in homozygous organoids are depicted in red, downregulated genes are blue. (C) Expression of genes associated with the ventral neural tube, dorsal neural tube, hindbrain, forebrain, WNT inhibition and WNT signalling. Values are scaled within rows. The heatmap shows CTRL samples generated with the AH017 line, and Hom (*PTCH1^−/−^*) samples generated with clones A2, B3 and H3. (D) Immunofluorescence of day 35 organoids with antibodies specific to the pan-neuronal marker NeuN (green) and ventral neural tube marker NKX2-2 (red). Nuclei are visualised in blue by Hoechst staining. Images are representative of four independent experiments. Scale bars: 250 μm.

The observed changes in cerebellar markers, organoid growth rate and morphology indicate significant changes in organoid development as a result of *PTCH1* loss. To investigate these further, 35-day-old control and *PTCH1-*mutant organoids were processed for bulk RNA sequencing. Using principal component analysis, we investigated transcriptome similarity and determined that samples clustered by genotype, with heterozygous samples locating closer to controls than to homozygous organoids (Fig. 3A). When we compared gene expression in control organoids with that in homozygous mutants, 4579 genes met the adjusted *P*-value cut-off (<0.05) of differential expression (Table S1). Interestingly, genes associated with the ventral neural tube (*SHH*, *FOXA1* and *FOXA2*) were among the most upregulated genes in homozygous organoids, whereas genes marking the dorsal neural tube (*WNT3A* and *TLX3*) were among the most downregulated (Fig. 3B). Further analysis revealed that ventral markers were highly expressed in homozygous organoids, whereas the expression of dorsal genes was lacking, compared to gene expression in control organoids, which showed an opposite pattern (Fig. 3C). Using immunofluorescence, we confirmed the ectopic expression of the ventral neural tube marker NKX2-2 in homozygous *PTCH1*^−/−^ mutant organoids but not in controls (Fig. 3D). These regions are suggestive of ventral patterning in homozygous organoids, similar to that seen in the developing neural tube (Briscoe and Ericson, 1999). When we analysed the expression of genes associated with regionalisation of the neural tube, *PTCH1*^−/−^ organoids displayed upregulation of genes related to the forebrain, including *NKX2-1*, *FOXG1* and *SIX3*, and downregulation of hindbrain markers (for example, *GBX2*, *ATOH1* and *LMX1A*), compared to their expression in controls (Fig. 3C). Consistent with a change in the neural differentiation trajectory, increased expression of the WNT antagonist *DKK1* and downregulation of the WNT readouts *AXIN2* and *LGR5* were observed, a process that is critical for the formation of forebrain structures (del Barco Barrantes et al., 2003; Glinka et al., 1998). Taken together, these findings indicate that the absence of PTCH1 function results in striking ventralisation and an anterior shift from mid-hindbrain to forebrain identity.

### The altered differentiation trajectory in *PTCH1*^−/−^ organoids is SHH signalling dependent

**Fig. 4.**
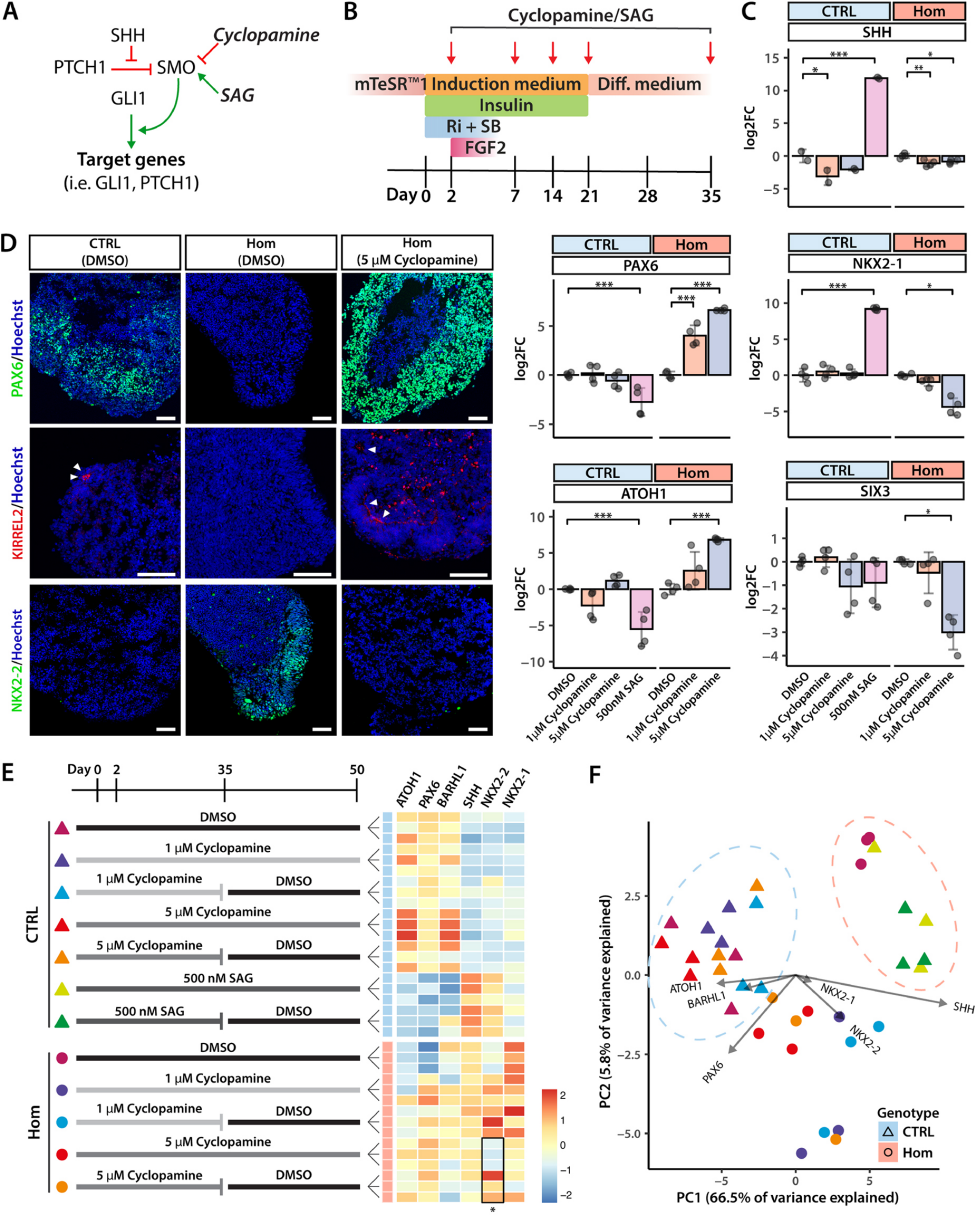
**SHH pathway inhibition rescues cerebellar marker expression.** (A) Simplified schematic of the SHH pathway with the effect of cyclopamine (red line, inhibitory) and SAG (green arrow, activating) on SMO. (B) Schematic of the experimental design to test the effect of SHH pathway inhibition on *PTCH1*^−/−^ cerebellar organoid differentiation. Ri, Rho kinase inhibitor Y-27632; SB, SB431542. (C) Changes in gene expression of day 35 organoids in different treatment conditions as measured by RT-qPCR and plotted as log_2_(fold change or FC). Expression is relative to that of the DMSO control condition of each line and normalised using *ACTB* and *GAPDH*. *n*=4 per condition from two differentiations. Error bars indicate s.d. Statistical significance was computed by one-way ANOVA: CTRL organoids {*PAX6* [*F*(3,12)=8.123, *P*=0.003], Dunnett's post hoc test (DMSO versus 500 nM SAG, *P*=0.004, 95% c.i. [0.9618, 4.5269]); *ATOH1* [*F*(3,12)=13.631, *P*<0.001], Dunnett's post hoc test (DMSO versus 500 nM SAG, *P*=0.001, 95% c.i. [2.4912, 8.5367]); *SHH* [*F*(3,12)=201.872, *P*<0.001], Dunnett's post hoc test (DMSO versus 1 µM cyclopamine, *P*=0.026, 95% c.i. [0.2447, 3.8296]; DMSO versus 500 nM SAG, *P*<0.001, 95% c.i. [−13.9309, −10.3460]); *NKX2-1* [*F*(3,12)=168.315, *P*<0.001], Dunnett's post hoc test (DMSO versus 500 nM SAG, *P*=0.001, 95% c.i. [−10.4973, −7.8783])}; Hom organoids {*ATOH1* [*F*(2,9)=19.421, *P*=0.001], Dunnett's post hoc test (DMSO versus 5 µM cyclopamine, *P*<0.001, 95% c.i. [−9.7165, −3.9305]); *PAX6* [*F*(2,9)=111.502, *P*<0.001], Dunnett's post hoc test (DMSO versus 1 µM cyclopamine, *P*<0.001, 95% c.i. [−5.2075, −2.8681]; DMSO versus 5 µM cyclopamine, *P*<0.001, 95% c.i. [−7.7988, −5.4595]); *SHH* [*F*(2,9)=9.029, *P*=0.007], Dunnett's post hoc test (DMSO versus 1 µM cyclopamine, *P*=0.005, 95% c.i. [0.4002, 1.8379]; DMSO versus 5 µM cyclopamine, *P*=0.02, 95% c.i. 0.1318, 1.5695]); *NKX2-1* [*F*(2,9)=35.711, *P*<0.001], Dunnett's post hoc test (DMSO versus 5 µM cyclopamine, *P*<0.001, 95% c.i. [2.9451, 5.79]); *SIX3* [*F*(2,9)=23.791, *P*<0.001], Dunnett's post hoc test (DMSO versus 5 µM cyclopamine: *P*<0.001, 95% c.i. [1.7809, 4.2339])}. *P<0.05; **P<0.01; ***P<0.001. (D) Immunofluorescence of day 35 organoids from different conditions with antibodies specific to PAX6 (green), KIRREL2 (red) or NKX2-2 (green). White arrowheads indicate the apical lumen of the cerebellar plate within the organoids, where typical staining of KIRREL2 can be seen. Nuclei are visualised in blue by Hoechst staining. Images are representative of four independent experiments. Scale bars: 100 μm. (E) Experimental design to test the effect of cyclopamine or SAG treatment cessation on cerebellar marker expression is depicted on the left. Heatmap showing the ΔCT values normalised to *ACTB* and *GAPDH* in each condition is shown on the right. *n*=3. The asterisk indicates statistical significance computed by two-tailed unpaired Student's *t*-test comparing treatment cessation with continued treatment [t(2)=−5.2331, *P*=0.03]. *P<0.05. Values are scaled within columns. (F) Principal component analysis showing different treatment and genotype conditions. The contribution of each gene to the principal components PC1 and PC2 is indicated by an arrow. The length of the arrow relates to the size of the contribution, indicating that *SHH* expression contributes the most to changes in PC1 and *PAX6* expression has the greatest effect on PC2. See also Fig. S8.

The role of SHH signalling in ventralisation of the neural tube has been described extensively (Patten and Placzek, 2000) and suggests that the altered differentiation trajectory in *PTCH1*^−/−^ organoids is likely caused by activated SHH signalling. We conducted experiments to either activate or repress SHH signalling in control and mutant organoids, with the aim of clarifying the role of SHH in cerebellar patterning early in development. First, we exposed control and homozygous organoids to a SHH signalling inhibitor and determined whether the ventralisation of *PTCH1*^−/−^ organoids could be blocked. During the first 35 days of differentiation, control and homozygous organoids were exposed to either DMSO or cyclopamine, which inhibits SHH signal transduction downstream of PTCH1 and at the level of SMO (Chen et al., 2002) (Fig. 4A,B). Cyclopamine treatment had only minor effects on gene expression in control organoids. In *PTCH1*^−/−^ organoids, cyclopamine rescued the expression of both *ATOH1* and *PAX6* in a dose-dependent manner, whereas it reduced the expression of *SHH* and the ventral forebrain genes *NKX2-1* and *SIX3* (Fig. 4C). Protein expression of PAX6 and NKX2-2, which are used *in vivo* as markers of dorsal and ventral neural tube specification, respectively, was determined using immunofluorescence (Fig. 4D). This showed a marked increase in the expression of PAX6 in homozygous organoids, surpassing PAX6 expression in control organoids. NKX2-2 expression was eliminated and KIRREL2 immunostaining was rescued, whereas expression of NeuN (RBFOX3), a pan-neuronal marker, remained unchanged (Fig. S8). These results confirm the change of the differentiation trajectory in *PTCH1*^−/−^ organoids to be SHH dependent and demonstrate how expression of cerebellar markers can be rescued in homozygous organoids by antagonizing SHH signalling.

Our data are consistent with the hypothesis that early, high-level SHH signalling results in a cell fate switch to a ventral forebrain-like identity marked by the expression of the NKX2-2 protein and *NKX2-1* gene that could be reversed by treatment with cyclopamine. This raises the question as to whether cyclopamine treatment of homozygous organoids could permanently rescue cerebellar differentiation. We therefore investigated the effects on gene expression of relief from SHH inhibition upon the continued culture of cyclopamine-treated organoids for another 15 days in the absence of the SMO inhibitor (Fig. 4E). We found that expression of the cerebellar GC markers *ATOH1*, *PAX6* and *BARHL1* at day 50 did not significantly decrease in organoids when cyclopamine treatment was discontinued after 35 days. Furthermore, *SHH* and *NKX2-1* expression remained at similar levels in organoids exposed to either the 35-day or 50-day cyclopamine treatment regimen. Most notably, *NKX2-*2 expression re-emerged after drug washout in homozygous organoids treated with 5 μM cyclopamine for 35 days. In other experiments, control organoids were treated with 500 nM of SMO agonist (SAG) to simulate enhanced SHH stimulation. SAG treatment significantly increased *SHH* and *NKX2-*1 expression and decreased mRNA levels of *ATOH1* and *PAX6* (Fig. 4C), similar to the changes seen in homozygous *PTCH1*^−/−^ organoids (Fig. 3C). These changes in gene expression persisted to at least day 50 even when SAG treatment was withdrawn after day 35.

We also performed principal component analysis of the ΔCT values generated by RT-qPCR to examine the transcriptional changes comprehensively. Control organoids exposed to SAG treatment projected towards *PTCH1*^−/−^ DMSO-treated organoids, suggesting similarity in gene expression (Fig. 4F). Cyclopamine treatment caused homozygous mutant organoids to cluster away from DMSO-treated homozygous organoids in principal component (PC) 1, depending on the dose of cyclopamine. Thus, SHH signalling appears to be the main driver of the gene expression profile differences between control and *PTCH1*^−/−^ homozygous organoids. This is corroborated by *SHH* being the main contributing gene in PC1, whereas the cerebellar GCP genes *ATOH1*, *PAX6* and *BARHL1* all act in the opposing direction. Taken together, these results show that high-level and early SHH signalling induces a ventral-forebrain differentiation trajectory in *PTCH1*^−/−^ homozygous organoids. This effect can be prevented by pharmacological SHH inhibition, resulting in lasting rescue of the dorsal hindbrain phenotype associated with cerebellar differentiation.

### *PTCH1*^+/−^ cerebellar organoids display tissue-specific effects of increased SHH signalling

**Fig. 5.**
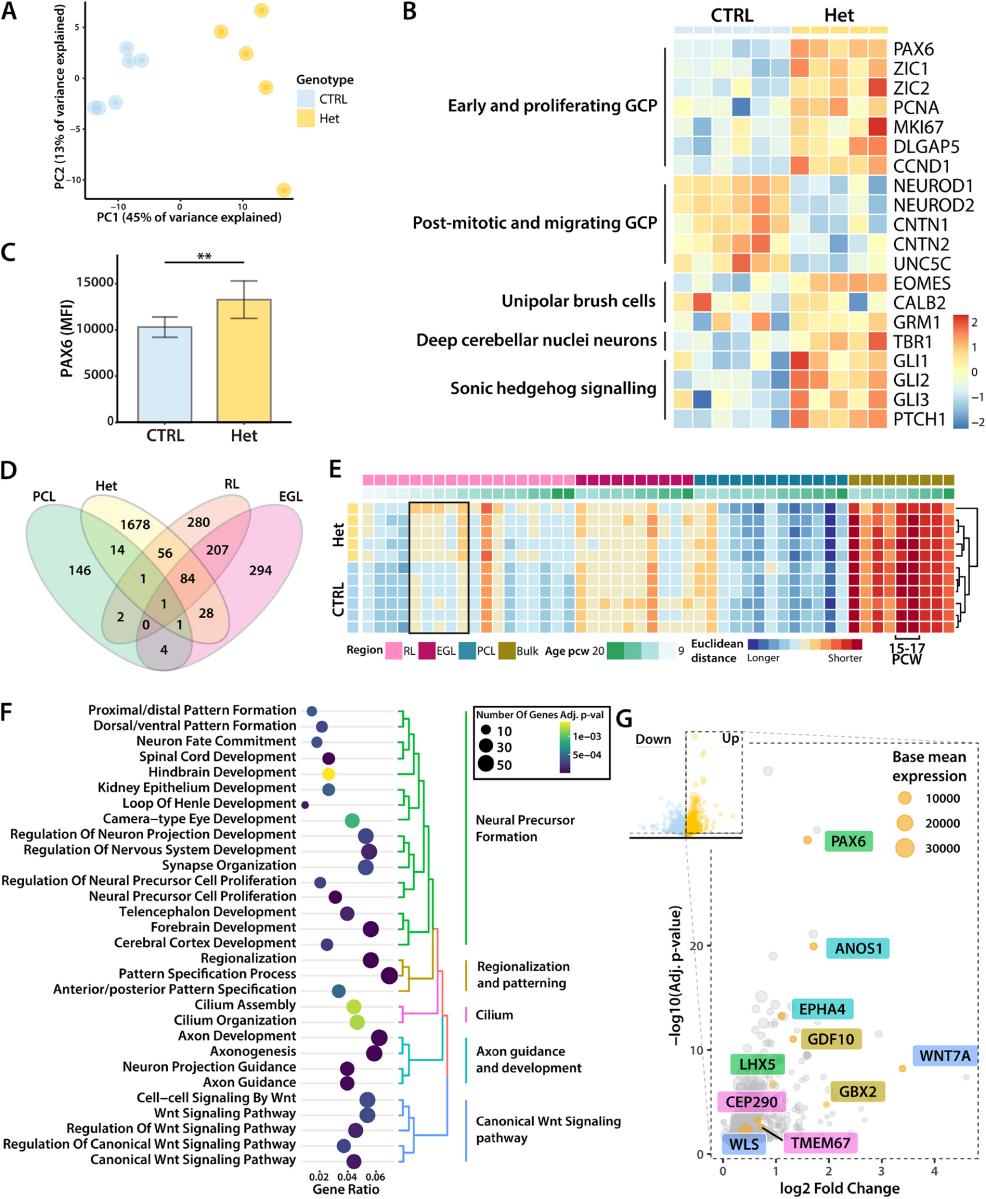
***PTCH1*-heterozygous cerebellar organoids display tissue-specific effects of increased SHH signalling.** (A) Principal component analysis using the 1000 most variable genes showing the separation of samples in principal components PC1 and PC2. Control (CTRL) samples were generated with the AH017 line, Het (*PTCH1^+/−^*) samples with clones A3, B2, B4 and C6. (B) Expression of genes associated with different stages of rhombic lip (RL) development and RL derivatives. Only differentially expressed genes are displayed. Values are scaled within rows. The heatmap shows control (CTRL) samples generated with the AH017 line, and Het (*PTCH1^+/−^*) samples generated with clones A3, B2, B4 and C6. (C) Bar graph of mean fluorescence intensity (MFI) values, measured by flow cytometry of PAX6-stained control (CTRL) and *PTCH1*^+/−^ (Het) organoids. A total of 50,000 cells per pool were measured. *n*=4. Error bars indicate s.d. Asterisks indicate statistical significance computed by two-tailed unpaired Student's *t*-test [t(5)=−5.1567, *P*=0.005]. **P<0.01. (D) Venn diagram displaying the overlap in differentially upregulated genes. (E) Heatmap showing the Euclidean distance measurements of organoid samples with laser capture microdissected (LCM) regional samples (Aldinger et al., 2021). Samples are organised by region and age. Measurements are scaled in rows. A shorter distance relates to a higher similarity in gene expression profile. The black outline indicates the difference in similarity to RL samples seen between heterozygous and wild-type organoids. In bulk samples, the time points with the highest similarity are marked. (F) Gene sets uniquely enriched in heterozygous organoids. The colour of the dots corresponds to the Benjamini–Hochberg adjusted *P*-value, and the size of the dots corresponds to the number of genes allocated to each gene set. (G) Volcano plot of selected representative genes associated with the top uniquely differentially expressed pathways in heterozygous organoids. The size of each data point corresponds to the base mean expression of the gene. EGL, external granule cell layer; GCP, granule cell progenitors; PCL, Purkinje cell layer; PCW, post conception weeks; RL, rhombic lip.

Next, we investigated transcriptomic changes in day 35 heterozygous *PTCH1*^+/−^ organoids. Because of sample dropout, we performed an additional round of sequencing to enhance statistical power. As before (Fig. 3A), PC1 (explaining 45% of transcriptome variance) separated samples by genotype (Fig. 5A). A total of 3821 genes reached the false discovery rate-adjusted *P*-value cut-off (<0.05) (Table S2). The SHH pathway genes *GLI1*, *GLI2*, *GLI3* and *PTCH1* were significantly upregulated in *PTCH1*^+/−^ organoids (Fig. 5B), which, taken together with the effect on growth (Fig. 2B), is consistent with increased SHH signal transduction. During cerebellar development, SHH signalling is most prominently known for inducing proliferation and expansion of the GCP population (Wechsler-Reya and Scott, 1999). In line with this, *PAX6* as well as *ZIC1* and *ZIC2*, two other GCP markers that are expressed throughout GC development (Aruga, 2004) and are thought to enhance SHH signalling (Brewster et al., 1998), were upregulated in heterozygous (*PTCH1^+/−^*) organoids. We also confirmed that the change in PAX6 expression was present at the protein level using flow cytometry of day 50 organoids, which showed a significantly higher mean fluorescence intensity (MFI) in *PTCH1*^+/−^ organoids compared to that in controls (*P*<0.01) (Fig. 5C). The increased expression of specific markers of the GC population was accompanied by the increased expression of genes associated with the cell cycle and proliferation (*PCNA*, *MKI67*, *TOP2A*, *DLGAP5* and *CCND1*) (Fig. 5B). Concurrently, expression of early post-mitotic markers (*NEUROD1* and *NEUROD2*) and migrating GC markers (*CNTN2*, *CNTN1*, *UNC5C*) (Ackerman et al., 1997; Miyata et al., 1999; Stoykova and Gruss, 1994) was decreased. Interestingly, the increased expression of GCP markers was accompanied by the elevation of *EOMES* and *TBR1* expression, marking the two other main cell types derived from the RL, unipolar brush cells and glutamatergic deep cerebellar nuclei, respectively (Fink et al., 2006; Mugnaini et al., 2011). There was no evidence for differential expression of genes (*CALB2*, *GRM1*) associated with more differentiated unipolar brush cells (Mugnaini et al., 2011). These results therefore suggest a broader effect of SHH-induced proliferation on RL derivatives. Taken together, our findings are consistent with an increased proportion of GCPs, concomitant with reduced differentiation, as a result of SHH signalling-induced proliferation in *PTCH1*^+/−^ heterozygous cerebellar organoids.

To investigate differences between control and *PTCH1*^+/−^ cerebellar organoids further, we sought to compare gene expression between specific regions and developmental stages of the cerebellum. To this end, organoid transcriptomes were compared with an established dataset generated from laser capture microdissected (LCM) samples of the developing human cerebellum (Aldinger et al., 2021). Differentially upregulated genes marking the RL, EGL and Purkinje cell layer as described in the original publication were compared to genes upregulated in *PTCH1*^+/−^ organoids. The most differentially expressed genes in the organoid samples overlapped with RL and EGL genes (Fig. 5D). Similarity with the different region-specific samples was then assessed by measuring Euclidean distances between LCM samples and organoid samples using the subset of spatial region-defining genes. Cerebellar organoids showed the most similarity with bulk samples at post-conception week (PCW) 15-17 (Fig. 5E). Among the LCM samples, organoids were most similar to RL samples at 15 PCW and to EGL samples at 17 PCW. Analysing distances of organoid samples to each respective region showed that *PTCH1^+/−^* heterozygous cerebellar organoids were more closely related to RL samples compared to control organoids. This suggests that RL derivatives make up a larger proportion of *PTCH1*^+/−^ organoids compared to controls.

In total, 1782 differentially expressed genes were unique to *PTCH1*^+/−^ heterozygous cerebellar organoids and not significantly altered or changed in the opposing direction in homozygous *PTCH1*^−/−^ organoids. Gene Ontology analysis was performed and revealed the enrichment of 154 gene sets, which were grouped into several main categories. Gene sets and key genes uniquely differentially expressed in heterozygous *PTCH1*^+/−^ organoids included those involved with cerebellar neuronal precursor formation (*PAX6* and *LHX5*), hindbrain and cerebellar regionalisation and patterning (*GDF10* and *GBX2*), axon development and guidance (*ANOS1* and *EPHA4*), the primary cilium (*CEP290* and *TMEM67*), and WNT signalling (*WLS* and *WNT7A*) (Fig. 5F,G). Taken together, these changes indicate that extensive and unique sets of genes and cerebellar developmental processes are regulated by distinct levels of SHH signalling in cerebellar organoids.

### *PTCH1*^+/−^ cerebellar organoids display relevant features of MB biology

**Fig. 6.**
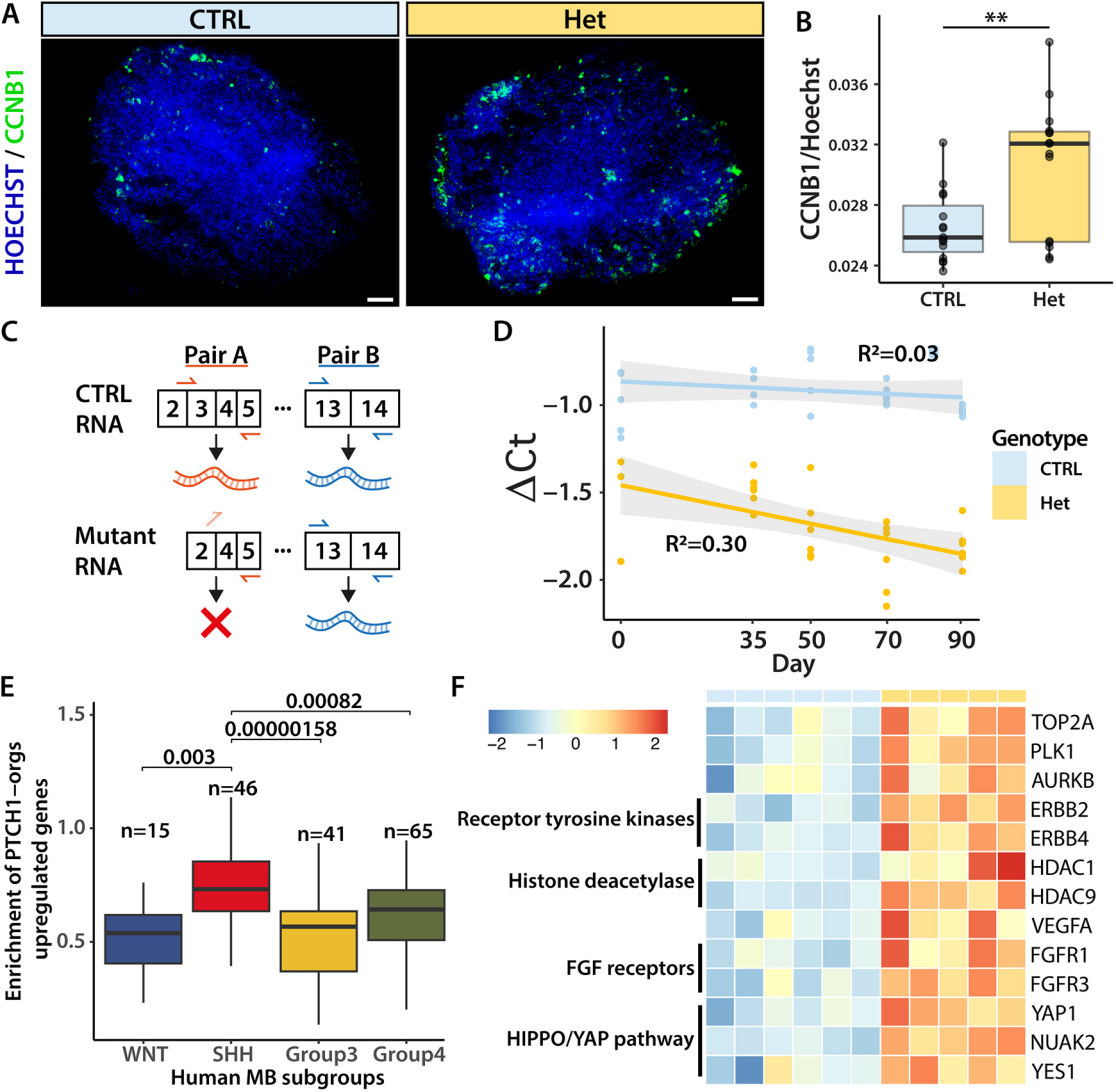
***PTCH1*-heterozygous organoids display relevant features for medulloblastoma biology.** (A) Immunofluorescence of day 35 control (CTRL) and *PTCH1^+/−^* (Het) organoids with antibodies specific to the proliferation marker CCNB1 (green). Nuclei are visualised with Hoechst. Scale bars: 100 μm. (B) Relative expression of CCNB1 normalised to Hoechst signals to correct for the number of nuclei in each organoid. Data points represent five different organoids from three differentiations. Error bars indicate s.d. Asterisks indicate statistical significance computed by two-tailed unpaired Student's *t*-test [t(21)=3.10, *P*=0.005]. **P<0.01. (C) Schematic of priming sites of primer pairs A and B. CTRL *PTCH1* mRNA allows amplification from both primer pairs, whereas the mutant mRNA only allows the amplification from pair B. (D) Dot plot of ΔCT values generated by normalising the expression of pair A to pair B. Linear regression line shows the relation of ΔCT with time. Lower values represent the lower relative expression of pair A, correlating with the respective expression of wild-type *PTCH1* mRNA. Data are from more than three biological replicates across separate differentiations. R^2^ values indicate the fit of the linear regression model. (E) Enrichment of genes upregulated in heterozygous *PTCH1*^+/−^ organoids (PTCH1-orgs) in subtypes of human medulloblastoma (MB). Genes are significantly enriched in SHH-MB samples. Box plots represent the interquartile range (IQR), the whiskers extend to values within 1.5 times the IQR from the quartiles, and the median is marked with a line. Statistical significance was computed using a two-tailed unpaired *t*-test with *P*-values adjusted by the Holm–Bonferroni method. See also Fig. S9. (F) Example genes associated with druggable targets that were found to be upregulated in heterozygous organoids. Values are scaled within rows. The heatmap shows CTRL samples generated with the AH017 line, and Het (*PTCH1^+/−^*) samples generated with clones A3, B2, B4 and C6.

The increased growth rate of *PTCH1*^+/−^ heterozygous organoids (Fig. 2) and higher expression of proliferation genes (Fig. 5) is consistent with findings in the *Ptch1*^+/−^ MB mouse model, which shows thickening of the EGL with increased and persisting proliferation of GCPs prior to developing MB (Oliver et al., 2005). To confirm increased proliferation on a protein level, we analysed the expression of cyclin B1 (CCNB1), marking cells transitioning from the G2 to M phase, across cerebellar organoids from three differentiations (Fig. 6A). Expression of CCNB1 was normalised to the Hoechst signal to correct for the number of nuclei in each organoid. We found that *PTCH1*^+/−^ heterozygous organoids expressed significantly more CCNB1 compared to controls (*P*=0.005) (Fig. 6A,B).

In addition to signs of increased proliferation, *Ptch1^+/−^* heterozygous mice display pre-neoplastic lesions with loss of heterozygosity that frequently precede malignant transformation (Oliver et al., 2005). To examine the possible loss of *PTCH1* expression in *PTCH1*^+/−^ heterozygous organoids, the relative expression of *PTCH1* was measured at five time points (days 0, 35, 50, 70 and 90) by RT-qPCR. Mutant *PTCH1* mRNA found in *PTCH1*^+/−^ heterozygous organoids does not contain exon 3 (Fig. 6C). Thus, by comparing the amplification of a region present in both the control mRNA and the gene-edited mutant mRNA with the amplification of exon 3, the relative prevalence of *PTCH1* control mRNA could be calculated. PCR amplification from primers spanning the exon–exon junction 2-3 and 5 only occurred from control *PTCH1* mRNA. Cycle threshold (CT) values generated from these primers were normalised to CT values produced using primers that amplify exons 13 to 14, which are present in both control and mutant *PTCH1* mRNA. Linear regression showed a statistically significant age-dependent decrease of *PTCH1* mRNA in heterozygous organoids (*P*=0.003) but not in control organoids (Fig. 6D), which might be indicative of loss of heterozygosity in the *PTCH1*^+/−^ organoids.

To investigate similarities with human MB, transcriptomes of *PTCH1^+/−^* organoids were compared to a publicly available dataset containing 167 human MB RNA-sequencing (RNA-seq) samples from the four different MB subgroups (Northcott et al., 2017). We found that upregulated genes in *PTCH1^+/−^* organoids were significantly enriched in SHH-MB compared to other MB subgroups (Fig. 6E)*.* In addition, CTRL and *PTCH1^+/−^* organoids were further clustered with the human MB RNA-seq dataset based on the normalised gene expression of subgroup-specific MB marker genes. In agreement with the results obtained by enrichment analysis, *PTCH1^+/−^* organoids clustered with SHH-MB samples, whereas control samples clustered with group 3 and 4 tumor samples (Fig. S9).

Taken together, our findings show that *PTCH1*^+/−^ heterozygous organoids display features associated with GCP proliferation and pre-neoplastic stages of SHH-MB, suggesting that the generated cerebellar organoids might be useful for the modelling of MB and as model systems to evaluate therapies. Interestingly, we found that various genes encoding known cancer targets were upregulated in *PTCH1*^+/−^ heterozygous cerebellar organoids (Fig. 6F), including MB-specific targets such as *TOP2A* (inhibited by etoposide) (Ruggiero et al., 2010; Su et al., 2022) as well as targets known in other types of cancer such as *PLK1* (inhibited by volasertib), *AURKB* (inhibited by barasertib) (Bavetsias and Linardopoulos, 2015), receptor tyrosine kinases such as *ERBB2* and *ERBB4* (inhibited by pertuzumab and afatinib) (Hynes and MacDonald, 2009), the histone deacetylases *HDAC1* and *HDAC9* (inhibited by vorinostat) (Perla et al., 2020), *VEGFA* (Apte et al., 2019), the fibroblast growth factor receptors *FGFR1* and *FGFR3* (Krook et al., 2021), and the YAP signalling components *YAP1*, *NUAK2* and *YES1* (Brodowska et al., 2014; Hamanaka et al., 2019). Together, these results suggest that *PTCH1*^+/−^ heterozygous organoids are useful for target discovery and to guide future studies of MB driver genes in *PTCH1*-mutant SHH-MB.

## DISCUSSION

In this study, heterozygous and homozygous LOF mutations of *PTCH1* were successfully introduced into iPSCs from a healthy donor using CRISPR/Cas9 gene editing. The mutation caused LOF of PTCH1 but did not affect pluripotency or proliferation of iPSCs. Directing these iPSC clones to cerebellar organoid differentiation demonstrated how early homozygous LOF of PTCH1 prevented cerebellar differentiation of iPSCs. In contrast, organoids heterozygous for PTCH1 LOF acquired a cerebellar identity, were more proliferative and contained an expanded glutamatergic lineage compared to control cerebellar organoids. Taken together, our findings show that developmental mechanisms in cerebellar organoids reflect *in vivo* processes of regionalisation and SHH signalling and offer new insight into early pathophysiological events of tumorigenesis. Importantly, the greater physiological relevance of the cerebellar organoid model allows the recapitulation of both major aspects of normal cerebellar development and pathological, pre-neoplastic developmental processes. Previously, such insights have required *in vivo* mouse models (Oliver et al., 2005), even when using human stem cell models (Huang et al., 2019; Susanto et al., 2020).

The pronounced effect of biallelic *PTCH1* LOF on cerebellar differentiation is in line with previous findings of mouse studies. In *Ptch1^−/−^* mice, neural tube closure fails and there is expansion of the ventral neural tube (Goodrich et al., 1997), which is consistent with the role of SHH signalling in orchestrating neuronal identity along the dorsoventral axis (Briscoe and Ericson, 1999; Martí and Bovolenta, 2002; Ribes and Briscoe, 2009). In vertebrates, secretion of SHH by the floor plate and notochord induces the expression of the NKX2-2 protein in adjacent neural progenitor domains, whereas it suppresses the expression of the PAX6 protein (Ericson et al., 1997). In line with this, *PTCH1^−/−^* organoids, which displayed high levels of *SHH* expression, were marked by upregulated *NKX2-2* expression and the absence of *PAX6* expression. Only discrete regions of the organoid expressed NKX2-2, suggesting that some level of spatial patterning occurs even *in vitro*. Furthermore, inhibition of SMO using cyclopamine rescued the expression of cerebellar markers. Therefore, in *PTCH1*^−/−^ homozygous organoids, the altered differentiation trajectory away from cerebellar identity and towards ventral fates results from hyperactivation of SHH signalling. The forebrain patterning defect is consistent with known SHH–WNT pathway interactions (Bertrand and Dahmane, 2006; Cederquist et al., 2019; Ulloa and Martí, 2010). Preventing early high-level SHH signalling had enduring effects on cerebellar marker expression, which is likely to reflect progressive cellular commitment to restricted, region-specific fates (Edlund and Jessell, 1999).

Increased SHH signalling in *PTCH1*^+/−^ organoids did not reach the threshold necessary to trigger a fate switch to ventral hindbrain identities and a cerebellar fate was retained. This enabled us to separate the effects of SHH signalling on fate specification from other SHH-regulated processes in the organoid and to gain insight into the effects of SHH signalling on the cellular constituents of the cerebellum. At an earlier stage of neural tube development when dorsoventral polarity is established, a high level of SHH signalling suppresses *PAX6* expression ventrally (Ericson et al., 1997). In our studies, the opposite is observed in *PTCH1*^+/−^ heterozygous cerebellar organoids, despite a higher level of SHH signalling. In the developing cerebellum, *PAX6* is expressed in RL derivatives and is involved in the early migration of GCPs from the outer EGL to the inner EGL (Engelkamp et al., 1999). The EGL therefore presents a unique developmental zone where *PAX6* expression persists in an environment in which high levels of SHH signalling drive GCP proliferation. The co-occurring increase of *PAX6*, *PTCH1*, *GLI1* and *GLI2* expression in *PTCH1*-heterozygous organoids suggests that these organoids contain EGL-like regions that show a tissue compartment-specific effect of SHH signalling.

As GCPs make up the majority of the RL lineage, our findings of increased expression of RL markers such as *PAX6* in *PTCH1*^+/−^ heterozygous organoids may be primarily the effect of an increased GCP population. However, increased expression of *WLS*, which marks the ventricular zone of the RL that contains RL neural stem cells (Haldipur et al., 2019; Yeung et al., 2014), may also support an effect of increased SHH signalling on broader RL development. Pathologies associated with aberrant human RL development manifest by cerebellar vermis hypoplasia (CVH) (Haldipur et al., 2019). WNT signalling has been implicated as a causative factor of CVH in ciliopathies (Lancaster et al., 2011), and *PTCH1^+/−^* organoids also displayed increased expression of WNT pathway genes, in contrast to the *PTCH1*^−/−^ homozygous organoids. A significant part of all CVH is caused by mutations in genes related to the primary cilium and the requirement of an intact primary cilium for SHH signalling is well described (Goetz and Anderson, 2010). The coordinated increase in gene expression related to the RL lineage, SHH pathway, WNT pathway and cilia in heterozygous organoids is further evidence for the early involvement of SHH in human cerebellar development by regulating WNT signalling.

Our cerebellar organoid model recapitulates the genetic defect in Gorlin syndrome (also known as nevoid basal cell carcinoma syndrome) (Gorlin and Goltz, 1960), which is caused by a germline *PTCH1*-heterozygous mutation and is associated with SHH-MB. Furthermore, the increased organoid growth, upregulation of cell cycle genes and increased expression of mitotic markers found in heterozygous organoids resemble the pre-neoplastic stage in *Ptch1^+/−^* mice (Oliver et al., 2005). *Ptch1^+/−^* mice exhibit a persisting hyperplastic EGL with pre-neoplastic lesions that have lost the wild-type allele. Similarly, wild-type *PTCH1* mRNA levels decreased in heterozygous organoids, suggesting loss of heterozygosity, which is frequently seen in both mouse models of MB (Ishida et al., 2010; Pazzaglia et al., 2006) and in human *PTCH1*-mutant MB (Northcott et al., 2017; Tamayo-Orrego et al., 2020). Studies have shown a clear association of SHH activation-induced DNA replication stress and elevated homologous recombination (Tamayo-Orrego et al., 2020). In line with these findings, heterozygous organoids displayed signs of loss of wild-type *PTCH1*, suggesting that our *in vitro* model could be used to gain further insights into these events. The similarity of the transcriptomes of *PTCH1-*heterozygous organoids and SHH-MB tumour samples further supports the validity of the organoid model and suggests that the changes observed in *PTCH1-*heterozygous organoids align with the SHH subgroup.

The presented study also has some limitations. Although we were able to generate and analyse the effect of the introduced mutation in multiple generated clones, thereby correcting for unidentified off-target CRISPR effects, we did not test the effect in different parental human iPSC lines. Furthermore, by using only one set of gRNAs, there is a risk of mutation site-specific effects that might not translate to the effects caused by other mutations in the *PTCH1* gene. Some caution is, therefore, appropriate when translating these results to other human iPSC lines as these might respond differently. Nevertheless, the robustness of the phenotype observed in our *PTCH1-*mutant organoids, combined with the strong correlation to phenotypes present in *Ptch1-*mutant mouse models and well-described early ventral-dorsal neural tube patterning, supports the conclusions drawn in our study.

We have shown that cerebellar organoid differentiation can accurately capture relevant human developmental phenotypes *in vitro* and our study complements findings from mouse models. This is especially relevant for MB for which findings in mouse models (Tamayo-Orrego et al., 2016) can diverge from large human genomic studies (Kool et al., 2008; Northcott et al., 2011; Skowron et al., 2021). Future investigations can apply the latest findings from these genome studies to cerebellar organoids to develop novel, human-specific models that are poised to aid the discovery of much-needed new treatments for patients with MB.

## MATERIALS AND METHODS

### iPSC line and culture

The previously described human iPSC line AH017-3 was used (Handel et al., 2016). iPSCs were cultured on hESC-qualified Matrigel matrix (Corning, 354277) in mTeSR1 medium (STEMCELL Technologies, 85850) or OXE8 medium [2 mM GlutaMAX (Gibco, 35050061), 0.1 μg/ml heparin (STEMCELL Technologies, 07980), 0.22 mM ascorbic acid, 15 mM HEPES pH 7.4, 100 ng/ml FGF2 (R&D Systems, 4114-TC-01M) and 2 ng/ml TGFβ (Peprotech, AF-100-21C) in advanced Dulbecco's modified Eagle medium/F12 (Gibco, 12634010)] (Vaughan-Jackson et al., 2021). The culture medium was changed daily and passaged using 0.5 mM EDTA in PBS. 10 μM Rho kinase inhibitor Y-27632 (Abcam, ab120129) was added to the culture medium during the first 24 h after passaging. Cells were kept in culture no longer than 5 weeks to minimise the chance of karyotypic changes. For each new experiment, a vial was used from the original master stock. This stock had passed quality control including SNP genotyping and mycoplasma testing.

### CRISPR-mediated gene editing

gRNAs were designed using CCtop (Stemmer et al., 2015). The exonic gRNA 5ʹ-GTGTTGTAGGAGCGCTTCTG-3ʹ and intronic gRNA 5ʹ-GATTTATCGTTTCTCGAGTT-3ʹ were picked based on their predicted specificity and efficiency. Predicted off-target sites are listed in Fig. S4.

CRISPR RNAs (crRNAs) and trans-activating CRISPR-RNAs (tracrRNAs) were heated to 95°C before slowly cooling to room temperature to generate crRNA/tracrRNA hybrids. The exonic and intronic targeting crRNA/tracrRNA hybrids were then mixed together in a 1:1 ratio. Ribonuclease proteins were made by complexing 44 µM crRNA/tracrRNA hybrids with 36 µM HiFi Cas9 Nuclease V3 (Integrated DNA Technologies, 1081060). 1 µl of ribonuclease proteins was mixed with 9 µl iPSC suspension (200,000 cells total) and the mixture was electroporated using the Neon Transfection System (MPK10096; HiTrans 1400V 20 ms width, one pulse). The cell pool was cultured to produce a CRISPR-pool stock and allow genomic DNA extraction (DNeasy kit, Qiagen, 69504). Successful CRISPR editing was visualised by PCR amplification of the target region (primers listed in Table S3). The mutant product was expected to be 243 bp shorter compared to the wild-type sequence. Monoclonal iPSC stocks were generated by single-cell plating on mouse embryonic feeders. Clones derived from a single cell were microscopically picked using a wide bore P200 tip. Selected clones were expanded to master stocks that were used for all downstream experiments.

### CRISPR clone quality control

DNA was extracted using the DNeasy kit and DNA was resuspended in 50 μl TE buffer. After isolation, genomic DNA was subjected to PCR amplification of the target region, followed by gel extraction (Monarch Gel Extraction Kit, New England Biolabs, T1020) and Sanger sequencing. Genomic DNA from the master stocks of each clone was submitted to a genotyping array to test for gross chromosomal aberrations (Illumina Infinium Global Screening Array-24 v3.0). Each clone was tested for mycoplasma using the MycoAlert PLUS Mycoplasma Detection Kit (Lonza, LT07-703). Pluripotency was measured by flow cytometry using the Tra-1-60 antibody (BioLegend, 330614, 1:100), an antibody against NANOG (Cell Signalling Technology, 5448S, 1:100) or isotype control antibodies (Alexa Fluor 488 IgM, 401617, or Alexa Fluor 647 IgG, 2985S, BioLegend, both 1:100).

### Differentiation into three germ layers

iPSCs of the different genotypes were differentiated into the three germ layers using the STEMdiff Trilineage Differentiation Kit (STEMCELL Technologies, 05230) following the manufacturer's protocol. iPSCs were seeded as single cells at the prescribed densities (ectoderm, 2×10^6^ cells/well; mesoderm, 0.5×10^6^ cells/well; endoderm, 2×10^6^ cells/well; and iPSC control, 0.5×10^6^ cells/well) on cover-slip-loaded six-well-plate wells. On day 1 after seeding, a full medium change was performed, changing to the respective lineage-specific medium, and the medium was changed daily afterwards. Mesoderm and endoderm progenitors were fixed on day 5 and ectoderm progenitors were fixed on day 7 using 2% paraformaldehyde (PFA), before washing with PBS and proceeding to immunostaining as described below.

### EdU proliferation assay

The proliferation of *PTCH1*^+/−^ and *PTCH1*^−/−^ iPSC clones was determined using the Click-iT Plus EdU Alexa Fluor 647 Flow Cytometry Assay Kit (Invitrogen, C10634). Proliferation was determined following the manufacturer's protocol and using 2 h of incubation with 10 μM of EdU. Proportions of cells in G0-G1, S and G2-M phase were determined by co-staining with Hoechst 33342 (Thermo Fisher Scientific, 62249) and analysis by flow cytometry. A total of 50,000 cells per sample were analysed.

### Cerebellar organoid differentiation

Cerebellar organoids were generated as described (van Essen et al., 2022; Nayler et al., 2021; Watson et al., 2018). In brief, iPSCs were detached using TrypLE Express Enzyme (1×) (Gibco, 12604013) and resuspended in induction medium [50% Iscove's modified Dulbecco's medium (Gibco, 31980022), 50% Ham's F-12 nutrient mix (Gibco, 31765027), 7 μg/ml insulin (Sigma-Aldrich, I1882), 5 mg/ml bovine serum albumin (Sigma-Aldrich, A3156-5G), 1% chemically defined lipid concentrate (Gibco, 11905031), 450 μM 1-thioglycerol (Sigma-Aldrich, M-6145), 15 μg/ml apo-transferrin (Sigma-Aldrich, T1147) and 1% penicillin/streptomycin (Gibco, 15140122)] containing 50µM Y-27632 and 10 µM SB431542 (Tocris, 1614/10). Embryoid bodies were made by seeding cells to 10,000 cells per well in a low-attachment V-bottom 96-well plate (Greiner Bio-one, 651970) and incubating them at 37°C with 5% CO_2_. Two days after seeding, cerebellar lineage induction was started by supplementing the medium with FGF2 (R&D Systems, 4114-TC-01M) to a final concentration of 50 ng/ml. Subsequent medium changes were performed weekly. On day 7, one-third of the medium was changed, and on other days, the medium was changed in full. On day 14, organoids were transferred to low-attachment 48-well plates (Greiner Bio-one, 677970). On day 21, the culture medium was changed to differentiation medium [Neurobasal medium (Gibco, 21103049), 1% GlutaMAX, 1% N2 supplement (Gibco, 17502048) and 1% penicillin/streptomycin]. Long-term culture from day 35 onward was performed on PTFE 0.4-µm pore size transwell membranes (Millicell, PICM0RG50). The number of organoids used was determined by the number required for different downstream analyses (see below).

For cyclopamine treatment, embryoid bodies of control and *PTCH1^−/−^* iPSCs were made as above. After 2 days, treatment was started and DMSO, 1 μM cyclopamine (Selleckchem, S1146), 5 μM cyclopamine or 500 nM SAG (Merck, 566660) was added to the medium upon medium change. At least 40 organoids were used for each treatment group for each replicate. Organoids were harvested after 35 days for RNA extraction and RT-qPCR or immunostaining.

### RT-qPCR

RNA was isolated using the RNeasy Plus Mini Kit (Qiagen, 74034) following the manufacturer's protocol. For iPSC samples, replicates extracted from different passages (∼2×10^6^ cells) were collected per clone. For organoid samples, RNA was extracted from pools of organoids. Typically, 40 organoids are required per replicate at day 21, 30 organoids at day 35, ten to 15 organoids at day 50, and one to three organoids at days 70 and 90 to yield sufficient RNA. The data shown in the figures were generated with the AH017 control line (CTRL), the *PTCH1^+/−^* A3 line (Het) and/or the *PTCH1^−/−^* H3 line (Hom). RNA was reverse transcribed into cDNA using the SuperScript III First-Strand Synthesis System (Invitrogen, 18080051). Standard curves to determine primer efficiency and optimal cDNA input were run prior to the experiment. RT-qPCR was performed using Fast SYBR Green Master Mix (Applied Biosystems, 4385612) on the Applied Biosystems StepOne Plus qPCR machine with primers designed using Primer3Plus (https://www.primer3plus.com). The primers used are listed in Table S3. Expression of the genes of interest (‘GOI’) was normalised to the expression of *ACTB* and *GAPDH* (‘REF’) to generate ΔCT values (ΔCT=CT^GOI^−CT^REF^). The average of these two ΔCT values was used for statistical analysis and visualisation. In comparisons with a control group, ΔΔCT values were calculated (ΔΔCT= ΔCT^test^−ΔCT^control average^). The expression of reference genes in each experiment was subjected to statistical testing for changes associated with the different conditions.

### Immunostaining

Organoids were fixed in 4% PFA and embedded in Optimal Cutting Temperature compound (Thermo Fisher Scientific, LAMB/OCT) for cryosectioning into 8 µm sections using a 5040 microtome (Bright Instruments, OTF5000). Cryosections were permeabilised in 0.3% Triton X-100 (Sigma-Aldrich, X100-100ML) in PBS (PBST) and blocked with 2% skim milk (Thermo Fisher Scientific, LP0031B) in PBST (blocking buffer). Primary antibodies (Table S4) diluted in blocking buffer were incubated overnight. Secondary antibodies (Table S5) diluted in blocking buffer were applied for 2 h. Nuclei were visualised with Hoechst 33342 and sections were mounted in anti-fading mounting solution (Vectashield, H-1000-10). Immunostained cryosections were imaged using a Zeiss Axioplan 2 widefield microscope or an Olympus FV1000 laser scanning confocal microscope. The data shown in the figures were generated with the AH017 control line (CTRL), the *PTCH1^+/−^* A3 line (Het) and/or the *PTCH1^−/−^* H3 line (Hom). The investigator was masked to the genotype when assessing outcomes.

### Flow cytometry

Organoids were washed once in PBS at room temperature and enzymatically digested using Neuron Isolation Enzyme (Thermo Fisher Scientific, 88285). Cells were washed in FACS buffer (1% bovine serum albumin in PBS) and fixed in 4% PFA. Permeabilisation was achieved using FACS buffer with 0.1% saponin (Thermo Fisher Scientific, A18820.22). Cells were incubated with PAX6 antibodies conjugated to allophycocyanin (Lightning Link Conjugation Kit, Abcam, ab201807) for 30 min. Cells were washed once more in FACS buffer and stained with Hoechst 33342. Flow cytometry was performed on the FACS Canto BD flow cytometer (BD Biosciences). The mean fluorescence intensity of 50,000 cells was measured and statistical analysis was performed as described below.

### RNA sequencing

RNA was isolated using the RNeasy Plus Micro Kit (Qiagen, 74034). RNA integrity was measured using the RNA 6000 Pico kit (Agilent, 5067-1513) on the 2100 Bioanalyzer instrument (Agilent). Samples that passed this quality measurement (RNA integrity number>9) were submitted for RNA sequencing. RNA concentration was determined using the Qubit 4 fluorometer (Invitrogen) following the manufacturer's protocol. A total of 400 ng per sample was submitted for library preparation. Library preparation and sequencing were performed by Novogene. Each library was submitted to 25 million paired-end reads (50 million total). Gene transcripts were quantified using Salmon 15.2 (https://combine-lab.github.io/salmon/) with GC content bias and sequencing length bias correction. Transcript quantifications were imported in R programming software using ‘tximeta’ (version 1.14.1, https://bioconductor.org/) (Ensembl *Homo sapiens* release 97). Differential gene expression was performed using DESeq2 (version 1.36.0; Love et al., 2014) and the ‘lfcshrink’ function that uses Bayesian shrinkage estimators for effect sizes (Zhu et al., 2019). Differentially expressed genes were identified as having a Benjamini–Hochberg adjusted *P*-value below 0.05. Gene expression was normalised using variance-stabilising transformation (VST) (‘vsn’ package version 3.64.0; https://bioconductor.org/). Principal component analysis was performed using ‘prcomp’ (‘stats’ package version 4.2.1; https://cran.r-project.org) using the top 1000 most variable genes and visualised using ‘ggplot2’ (version 3.3.6; https://cran.r-project.org). Gene expression was visualised using ‘pheatmap’ (version 1.0.12; https://cran.r-project.org). Gene set variation analysis (GSVA) was performed using the GSVA package (version 1.44.2; https://bioconductor.org/). Gene Ontology analysis was performed using the Clusterprofiler package (version 4.4.4; https://bioconductor.org/). Single-sample gene set enrichment analysis was performed using the ‘ssgsea’ method from the GSVA package (version 1.48.3). A gene set corresponding to upregulated genes in the *PTCH1-*heterozygous organoids [adjusted *P*-value<0.05, log_2_(fold change)>1] was subjected to enrichment analysis using a cohort of 167 human MB RNA-seq profiles (filtered by differentially expressed genes) classified by molecular subgroup (Northcott et al., 2017). *PTCH1^+/−^* heterozygous and CTRL organoids were further clustered with the human MB RNA-seq dataset using an unsupervised hierarchical clustering method based on the normalised gene expression of 917 subgroup-specific marker genes (curated from 1121 previously published human MB microarray profiles) (Cavalli et al., 2017). All data and code are available on https://github.com/mxvssn.

### Statistics

Statistical analysis was performed in R programming software. The normality of the data was assessed using quantile–quantile plots of the residuals. Sample size was determined based on pilot experiments and previous experience. Statistical difference between more than two groups was determined using one-way ANOVA with Dunnett's or Tukey's post hoc test. Statistical differences between two groups were performed by two-tailed unpaired Student's *t*-test. *P*-values were corrected for multiple testing using the Benjamini–Hochberg procedure. An adjusted *P*-value below 0.05 was considered significant. Error bars represent standard deviation (s.d.) unless otherwise indicated.

## Supplementary Material

10.1242/dmm.050323_sup1 Supplementary information

Table S1. Differentially expressed genes in homozygous clones

Table S2. Differentially expressed genes in heterozygous clones

## References

[DMM050323C1] Ackerman, S. L., Kozak, L. P., Przyborski, S. A., Rund, L. A., Boyer, B. B. and Knowles, B. B. (1997). The mouse rostral cerebellar malformation gene encodes an UNC-5-like protein. *Nature* 386, 838-842. 10.1038/386838a0 9126743

[DMM050323C2] Aguilar, A., Meunier, A., Strehl, L., Martinovic, J., Bonniere, M., Attie-Bitach, T., Encha-Razavi, F. and Spassky, N. (2012). Analysis of human samples reveals impaired SHH-dependent cerebellar development in Joubert syndrome/Meckel syndrome. *Proc. Natl. Acad. Sci. USA* 109, 16951-16956. 10.1073/pnas.1201408109 23027964 PMC3479472

[DMM050323C3] Aldinger, K. A., Thomson, Z., Phelps, I. G., Haldipur, P., Deng, M., Timms, A. E., Hirano, M., Santpere, G., Roco, C., Rosenberg, A. B. et al. (2021). Spatial and cell type transcriptional landscape of human cerebellar development. *Nat. Neurosci.* 24, 1163-1175. 10.1038/s41593-021-00872-y 34140698 PMC8338761

[DMM050323C4] Apte, R. S., Chen, D. S. and Ferrara, N. (2019). VEGF in signaling and disease: beyond discovery and development. *Cell* 176, 1248-1264. 10.1016/j.cell.2019.01.021 30849371 PMC6410740

[DMM050323C5] Aruga, J. (2004). The role of Zic genes in neural development. *Mol. Cell. Neurosci.* 26, 205-221. 10.1016/j.mcn.2004.01.004 15207846

[DMM050323C6] Bavetsias, V. and Linardopoulos, S. (2015). Aurora kinase inhibitors: current status and outlook. *Front. Oncol.* 5, 278. 10.3389/fonc.2015.00278 26734566 PMC4685048

[DMM050323C7] Ben-David, U., Ha, G., Tseng, Y.-Y., Greenwald, N. F., Oh, C., Shih, J., McFarland, J. M., Wong, B., Boehm, J. S., Beroukhim, R. et al. (2017). Patient-derived xenografts undergo mouse-specific tumor evolution. *Nat. Genet.* 49, 1567-1575. 10.1038/ng.3967 28991255 PMC5659952

[DMM050323C8] Bertrand, N. and Dahmane, N. (2006). Sonic hedgehog signaling in forebrain development and its interactions with pathways that modify its effects. *Trends Cell Biol.* 16, 597-605. 10.1016/j.tcb.2006.09.007 17030124

[DMM050323C9] Bian, S., Repic, M., Guo, Z., Kavirayani, A., Burkard, T., Bagley, J. A., Krauditsch, C. and Knoblich, J. A. (2018). Genetically engineered cerebral organoids model brain tumor formation. *Nat. Methods* 15, 631-639. 10.1038/s41592-018-0070-7 30038414 PMC6071863

[DMM050323C10] Blassberg, R. and Jacob, J. (2017). Lipid metabolism fattens up hedgehog signaling. *BMC Biol.* 15, 95. 10.1186/s12915-017-0442-y 29073896 PMC5659038

[DMM050323C11] Bouaoun, L., Sonkin, D., Ardin, M., Hollstein, M., Byrnes, G., Zavadil, J. and Olivier, M. (2016). TP53 variations in human cancers: new lessons from the IARC TP53 database and genomics data. *Hum. Mutat.* 37, 865-876. 10.1002/humu.23035 27328919

[DMM050323C12] Brewster, R., Lee, J. and Ruiz i Altaba, A. (1998). Gli/Zic factors pattern the neural plate by defining domains of cell differentiation. *Nature* 393, 579-583. 10.1038/31242 9634234

[DMM050323C13] Briscoe, J. and Ericson, J. (1999). The specification of neuronal identity by graded Sonic Hedgehog signalling. *Semin. Cell Dev. Biol.* 10, 353-362. 10.1006/scdb.1999.0295 10441550

[DMM050323C14] Brodowska, K., Al-Moujahed, A., Marmalidou, A., Meyer Zu Horste, M., Cichy, J., Miller, J. W., Gragoudas, E. and Vavvas, D. G. (2014). The clinically used photosensitizer Verteporfin (VP) inhibits YAP-TEAD and human retinoblastoma cell growth in vitro without light activation. *Exp. Eye Res.* 124, 67-73. 10.1016/j.exer.2014.04.011 24837142 PMC4135181

[DMM050323C15] Cavalli, F. M. G., Remke, M., Rampasek, L., Peacock, J., Shih, D. J. H., Luu, B., Garzia, L., Torchia, J., Nor, C., Morrissy, A. S. et al. (2017). Intertumoral heterogeneity within Medulloblastoma subgroups. *Cancer Cell* 31, 737-754.e6. 10.1016/j.ccell.2017.05.005 28609654 PMC6163053

[DMM050323C16] Cederquist, G. Y., Asciolla, J. J., Tchieu, J., Walsh, R. M., Cornacchia, D., Resh, M. D. and Studer, L. (2019). Specification of positional identity in forebrain organoids. *Nat. Biotechnol.* 37, 436-444. 10.1038/s41587-019-0085-3 30936566 PMC6447454

[DMM050323C17] Chen, J. K., Taipale, J., Cooper, M. K. and Beachy, P. A. (2002). Inhibition of Hedgehog signaling by direct binding of cyclopamine to Smoothened. *Genes Dev.* 16, 2743-2748. 10.1101/gad.1025302 12414725 PMC187469

[DMM050323C18] Corbit, K. C., Aanstad, P., Singla, V., Norman, A. R., Stainier, D. Y. R. and Reiter, J. F. (2005). Vertebrate Smoothened functions at the primary cilium. *Nature* 437, 1018-1021. 10.1038/nature04117 16136078

[DMM050323C19] Corcoran, R. B. and Scott, M. P. (2006). Oxysterols stimulate Sonic hedgehog signal transduction and proliferation of medulloblastoma cells. *Proc. Natl. Acad. Sci. USA* 103, 8408-8413. 10.1073/pnas.0602852103 16707575 PMC1462959

[DMM050323C20] Dahmane, N. and Ruiz i Altaba, A. (1999). Sonic hedgehog regulates the growth and patterning of the cerebellum. *Development* 126, 3089-3100. 10.1242/dev.126.14.3089 10375501

[DMM050323C21] del Barco Barrantes, I., Davidson, G., Gröne, H.-J., Westphal, H. and Niehrs, C. (2003). Dkk1 and noggin cooperate in mammalian head induction. *Genes Dev.* 17, 2239-2244. 10.1101/gad.269103 12952897 PMC196461

[DMM050323C22] Denef, N., Neubüser, D., Perez, L. and Cohen, S. M. (2000). Hedgehog induces opposite changes in turnover and subcellular localization of patched and smoothened. *Cell* 102, 521-531. 10.1016/S0092-8674(00)00056-8 10966113

[DMM050323C23] Dwyer, J. R., Sever, N., Carlson, M., Nelson, S. F., Beachy, P. A. and Parhami, F. (2007). Oxysterols are novel activators of the hedgehog signaling pathway in pluripotent mesenchymal cells. *J. Biol. Chem.* 282, 8959-8968. 10.1074/jbc.M611741200 17200122

[DMM050323C24] Edlund, T. and Jessell, T. M. (1999). Progression from extrinsic to intrinsic signaling in cell fate specification: a view from the nervous system. *Cell* 96, 211-224. 10.1016/S0092-8674(00)80561-9 9988216

[DMM050323C25] Engelkamp, D., Rashbass, P., Seawright, A. and van Heyningen, V. (1999). Role of Pax6 in development of the cerebellar system. *Development* 126, 3585-3596. 10.1242/dev.126.16.3585 10409504

[DMM050323C26] Ericson, J., Rashbass, P., Schedl, A., Brenner-Morton, S., Kawakami, A., van Heyningen, V., Jessell, T. M. and Briscoe, J. (1997). Pax6 controls progenitor cell identity and neuronal fate in response to graded Shh signaling. *Cell* 90, 169-180. 10.1016/S0092-8674(00)80323-2 9230312

[DMM050323C27] Fink, A. J., Englund, C., Daza, R. A. M., Pham, D., Lau, C., Nivison, M., Kowalczyk, T. and Hevner, R. F. (2006). Development of the deep cerebellar nuclei: transcription factors and cell migration from the rhombic lip. *J. Neurosci.* 26, 3066-3076. 10.1523/JNEUROSCI.5203-05.2006 16540585 PMC6673970

[DMM050323C28] Fuccillo, M., Joyner, A. L. and Fishell, G. (2006). Morphogen to mitogen: the multiple roles of hedgehog signalling in vertebrate neural development. *Nat. Rev. Neurosci.* 7, 772-783. 10.1038/nrn1990 16988653

[DMM050323C29] Garcia-Lopez, J., Kumar, R., Smith, K. S. and Northcott, P. A. (2021). Deconstructing Sonic Hedgehog medulloblastoma: molecular subtypes, drivers, and beyond. *Trends Genet.* 37, 235-250. 10.1016/j.tig.2020.11.001 33272592

[DMM050323C30] Glinka, A., Wu, W., Delius, H., Monaghan, A. P., Blumenstock, C. and Niehrs, C. (1998). Dickkopf-1 is a member of a new family of secreted proteins and functions in head induction. *Nature* 391, 357-362. 10.1038/34848 9450748

[DMM050323C31] Goetz, S. C. and Anderson, K. V. (2010). The primary cilium: a signalling centre during vertebrate development. *Nat. Rev. Genet.* 11, 331-344. 10.1038/nrg2774 20395968 PMC3121168

[DMM050323C32] Goodrich, L. V., Johnson, R. L., Milenkovic, L., McMahon, J. A. and Scott, M. P. (1996). Conservation of the hedgehog/patched signaling pathway from flies to mice: Induction of a mouse patched gene by Hedgehog. *Genes Dev.* 10, 301-312. 10.1101/gad.10.3.301 8595881

[DMM050323C33] Goodrich, L. V., Milenković, L., Higgins, K. M. and Scott, M. P. (1997). Altered neural cell fates and medulloblastoma in mouse patched mutants. *Science* 277, 1109-1113. 10.1126/science.277.5329.1109 9262482

[DMM050323C34] Gorlin, R. J. and Goltz, R. W. (1960). Multiple nevoid basal-cell epithelioma, Jaw Cysts and Bifid Rib. *N. Engl. J. Med.* 262, 908-912. 10.1056/NEJM196005052621803 13851319

[DMM050323C35] Haldipur, P., Aldinger, K. A., Bernardo, S., Deng, M., Timms, A. E., Overman, L. M., Winter, C., Lisgo, S. N., Razavi, S. N., Silvestri, E. et al. (2019). Spatiotemporal expansion of primary progenitor zones in the developing human cerebellum. *Science* 366, 454-460. 10.1126/science.aax7526 31624095 PMC6897295

[DMM050323C36] Hamanaka, N., Nakanishi, Y., Mizuno, T., Horiguchi-Takei, K., Akiyama, N., Tanimura, H., Hasegawa, M., Satoh, Y., Tachibana, Y., Fujii, T. et al. (2019). YES1 is a targetable oncogene in cancers harboring YES1 gene amplification. *Cancer Res.* 79, 5734-5745. 10.1158/0008-5472.CAN-18-3376 31391186

[DMM050323C37] Handel, A. E., Chintawar, S., Lalic, T., Whiteley, E., Vowles, J., Giustacchini, A., Argoud, K., Sopp, P., Nakanishi, M., Bowden, R. et al. (2016). Assessing similarity to primary tissue and cortical layer identity in induced pluripotent stem cell-derived cortical neurons through single-cell transcriptomics. *Hum. Mol. Genet.* 25, 989-1000. 10.1093/hmg/ddv637 26740550 PMC4754051

[DMM050323C38] Hovestadt, V., Smith, K. S., Bihannic, L., Filbin, M. G., Shaw, M. K. L., Baumgartner, A., DeWitt, J. C., Groves, A., Mayr, L., Weisman, H. R. et al. (2019). Resolving medulloblastoma cellular architecture by single-cell genomics. *Nature* 572, 74-79. 10.1038/s41586-019-1434-6 31341285 PMC6754173

[DMM050323C39] Huang, M., Tailor, J., Zhen, Q., Gillmor, A. H., Miller, M. L., Weishaupt, H., Chen, J., Zheng, T., Nash, E. K., McHenry, L. K. et al. (2019). Engineering genetic predisposition in human neuroepithelial stem cells recapitulates medulloblastoma tumorigenesis. *Cell Stem Cell* 25, 433-446.e7. 10.1016/j.stem.2019.05.013 31204176 PMC6731167

[DMM050323C40] Hui, C.-C. and Angers, S. (2011). Gli proteins in development and disease. *Annu. Rev. Cell Dev. Biol.* 27, 513-537. 10.1146/annurev-cellbio-092910-154048 21801010

[DMM050323C41] Hynes, N. E. and MacDonald, G. (2009). ErbB receptors and signaling pathways in cancer. *Curr. Opin. Cell Biol.* 21, 177-184. 10.1016/j.ceb.2008.12.010 19208461

[DMM050323C42] Ingham, P. W., Nakano, Y. and Seger, C. (2011). Mechanisms and functions of Hedgehog signalling across the metazoa. *Nat. Rev. Genet.* 12, 393-406. 10.1038/nrg2984 21502959

[DMM050323C43] Ishida, Y., Takabatake, T., Kakinuma, S., Doi, K., Yamauchi, K., Kaminishi, M., Kito, S., Ohta, Y., Amasaki, Y., Moritake, H. et al. (2010). Genomic and gene expression signatures of radiation in medulloblastomas after low-dose irradiation in Ptch1 heterozygous mice. *Carcinogenesis* 31, 1694-1701. 10.1093/carcin/bgq145 20616149

[DMM050323C44] Jacks, T., Remington, L., Williams, B. O., Schmitt, E. M., Halachmi, S., Bronson, R. T. and Weinberg, R. A. (1994). Tumor spectrum analysis in p53-mutant mice. *Curr. Biol.* 4, 1-7. 10.1016/S0960-9822(00)00002-6 7922305

[DMM050323C45] Jackson, T. W., Bendfeldt, G. A., Beam, K. A., Rock, K. D. and Belcher, S. M. (2020). Heterozygous mutation of sonic hedgehog receptor (Ptch1) drives cerebellar overgrowth and sex-specifically alters hippocampal and cortical layer structure, activity, and social behavior in female mice. *Neurotoxicol. Teratol.* 78, 106866. 10.1016/j.ntt.2020.106866 32113901 PMC8018584

[DMM050323C46] Khaliullina, H., Panáková, D., Eugster, C., Riedel, F., Carvalho, M. and Eaton, S. (2009). Patched regulates Smoothened trafficking using lipoprotein-derived lipids. *Development* 136, 4111-4121. 10.1242/dev.041392 19906846

[DMM050323C47] Kool, M., Koster, J., Bunt, J., Hasselt, N. E., Lakeman, A., van Sluis, P., Troost, D., Meeteren, N. S., Caron, H. N., Cloos, J. et al. (2008). Integrated genomics identifies five medulloblastoma subtypes with distinct genetic profiles, pathway signatures and clinicopathological features. *PLoS ONE* 3, e3088. 10.1371/journal.pone.0003088 18769486 PMC2518524

[DMM050323C48] Krook, M. A., Reeser, J. W., Ernst, G., Barker, H., Wilberding, M., Li, G., Chen, H.-Z. and Roychowdhury, S. (2021). Fibroblast growth factor receptors in cancer: genetic alterations, diagnostics, therapeutic targets and mechanisms of resistance. *Br. J. Cancer* 124, 880-892. 10.1038/s41416-020-01157-0 33268819 PMC7921129

[DMM050323C49] Lago, C., Gianesello, M., Santomaso, L., Leva, G., Ballabio, C., Anderle, M., Antonica, F. and Tiberi, L. (2023). Medulloblastoma and high-grade glioma organoids for drug screening, lineage tracing, co-culture and in vivo assay. *Nat. Protoc.* 18, 2143-2180. 10.1038/s41596-023-00839-2 37248391

[DMM050323C50] Lancaster, M. A., Gopal, D. J., Kim, J., Saleem, S. N., Silhavy, J. L., Louie, C. M., Thacker, B. E., Williams, Y., Zaki, M. S. and Gleeson, J. G. (2011). Defective Wnt-dependent cerebellar midline fusion in a mouse model of Joubert syndrome. *Nat. Med.* 17, 726-731. 10.1038/nm.2380 21623382 PMC3110639

[DMM050323C51] Lee, J., Platt, K. A., Censullo, P. and Ruiz i Altaba, A. (1997). Gli1 is a target of Sonic hedgehog that induces ventral neural tube development. *Development* 124, 2537-2552. 10.1242/dev.124.13.2537 9216996

[DMM050323C52] Leto, K., Arancillo, M., Becker, E. B. E., Buffo, A., Chiang, C., Ding, B., Dobyns, W. B., Dusart, I., Haldipur, P., Hatten, M. E. et al. (2016). Consensus paper: cerebellar development. *Cerebellum* 15, 789-828. 10.1007/s12311-015-0724-2 26439486 PMC4846577

[DMM050323C53] Liu, A. and Joyner, A. L. (2001). Early anterior/posterior patterning of the midbrain and cerebellum. *Annu. Rev. Neurosci.* 24, 869-896. 10.1146/annurev.neuro.24.1.869 11520921

[DMM050323C54] Love, M. I., Huber, W. and Anders, S. (2014). Moderated estimation of fold change and dispersion for RNA-seq data with DESeq2. *Genome Biol.* 15, 550. 10.1186/s13059-014-0550-8 25516281 PMC4302049

[DMM050323C55] Martí, E. and Bovolenta, P. (2002). Sonic hedgehog in CNS development: one signal, multiple outputs. *Trends Neurosci.* 25, 89-96. 10.1016/S0166-2236(02)02062-3 11814561

[DMM050323C56] Miyata, T., Maeda, T. and Lee, J. E. (1999). NeuroD is required for differentiation of the granule cells in the cerebellum and hippocampus. *Genes Dev.* 13, 1647-1652. 10.1101/gad.13.13.1647 10398678 PMC316850

[DMM050323C57] Mugnaini, E., Sekerková, G. and Martina, M. (2011). The unipolar brush cell: a remarkable neuron finally receiving deserved attention. *Brain Res. Rev.* 66, 220-245. 10.1016/j.brainresrev.2010.10.001 20937306 PMC3030675

[DMM050323C58] Muguruma, K., Nishiyama, A., Kawakami, H., Hashimoto, K. and Sasai, Y. (2015). Self-organization of polarized cerebellar tissue in 3D culture of human pluripotent stem cells. *Cell Rep.* 10, 537-550. 10.1016/j.celrep.2014.12.051 25640179

[DMM050323C59] Nachtergaele, S., Mydock, L. K., Krishnan, K., Rammohan, J., Schlesinger, P. H., Covey, D. F. and Rohatgi, R. (2012). Oxysterols are allosteric activators of the oncoprotein Smoothened. *Nat. Chem. Biol.* 8, 211-220. 10.1038/nchembio.765 22231273 PMC3262054

[DMM050323C60] Nayler, S., Agarwal, D., Curion, F., Bowden, R. and Becker, E. B. E. (2021). High-resolution transcriptional landscape of xeno-free human induced pluripotent stem cell-derived cerebellar organoids. *Sci. Rep.* 11, 12959. 10.1038/s41598-021-91846-4 34155230 PMC8217544

[DMM050323C61] Northcott, P. A., Korshunov, A., Witt, H., Hielscher, T., Eberhart, C. G., Mack, S., Bouffet, E., Clifford, S. C., Hawkins, C. E., French, P. et al. (2011). Medulloblastoma comprises four distinct molecular variants. *J. Clin. Oncol.* 29, 1408-1414. 10.1200/JCO.2009.27.4324 20823417 PMC4874239

[DMM050323C62] Northcott, P. A., Buchhalter, I., Morrissy, A. S., Hovestadt, V., Weischenfeldt, J., Ehrenberger, T., Gröbner, S., Segura-Wang, M., Zichner, T., Rudneva, V. A. et al. (2017). The whole-genome landscape of medulloblastoma subtypes. *Nature* 547, 311-317. 10.1038/nature22973 28726821 PMC5905700

[DMM050323C63] Northcott, P. A., Robinson, G. W., Kratz, C. P., Mabbott, D. J., Pomeroy, S. L., Clifford, S. C., Rutkowski, S., Ellison, D. W., Malkin, D., Taylor, M. D. et al. (2019). Medulloblastoma. *Nat. Rev. Dis. Primers* 5, 11. 10.1038/s41572-019-0063-6 30765705

[DMM050323C64] Ogawa, J., Pao, G. M., Shokhirev, M. N. and Verma, I. M. (2018). Glioblastoma model using human cerebral organoids. *Cell Rep.* 23, 1220-1229. 10.1016/j.celrep.2018.03.105 29694897 PMC6892608

[DMM050323C65] Oliver, T. G., Read, T. A., Kessler, J. D., Mehmeti, A., Wells, J. F., Huynh, T. T. T., Lin, S. M. and Wechsler-Reya, R. J. (2005). Loss of patched and disruption of granule cell development in a pre-neoplastic stage of medulloblastoma. *Development* 132, 2425-2439. 10.1242/dev.01793 15843415

[DMM050323C66] Ostrom, Q. T., Gittleman, H., Truitt, G., Boscia, A., Kruchko, C. and Barnholtz-Sloan, J. S. (2018). CBTRUS statistical report: primary brain and other central nervous system tumors diagnosed in the United States in 2011-2015. *Neuro Oncol.* 20, iv1-iv86. 10.1093/neuonc/noy131 30445539 PMC6129949

[DMM050323C67] Patten, I. and Placzek, M. (2000). The role of Sonic hedgehog in neural tube patterning. *Cell. Mol. Life Sci.* 57, 1695-1708. 10.1007/PL00000652 11130176 PMC11146859

[DMM050323C68] Pazzaglia, S., Tanori, M., Mancuso, M., Gessi, M., Pasquali, E., Leonardi, S., Oliva, M. A., Rebessi, S., Di Majo, V., Covelli, V. et al. (2006). Two-hit model for progression of medulloblastoma preneoplasia in Patched heterozygous mice. *Oncogene* 25, 5575-5580. 10.1038/sj.onc.1209544 16636673

[DMM050323C69] Perla, A., Fratini, L., Cardoso, P. S., Nör, C., Brunetto, A. T., Brunetto, A. L., de Farias, C. B., Jaeger, M. and Roesler, R. (2020). Histone deacetylase inhibitors in pediatric brain cancers: biological activities and therapeutic potential. *Front. Cell Dev. Biol.* 8, 546. 10.3389/fcell.2020.00546 32754588 PMC7365945

[DMM050323C70] Ribes, V. and Briscoe, J. (2009). Establishing and interpreting graded Sonic Hedgehog signaling during vertebrate neural tube patterning: the role of negative feedback. *Cold Spring Harb. Perspect. Biol.* 1, a002014. 10.1101/cshperspect.a002014 20066087 PMC2742090

[DMM050323C71] Ruggiero, A., Rizzo, D., Attinà, G., Lazzareschi, I., Mastrangelo, S., Maurizi, P., Migliorati, R., Bertolini, P., Pastore, M., Colosimo, C. et al. (2010). Phase I study of temozolomide combined with oral etoposide in children with recurrent or progressive medulloblastoma. *Eur. J. Cancer* 46, 2943-2949. 10.1016/j.ejca.2010.05.016 20538454

[DMM050323C72] Ruiz i Altaba, A., Palma, V. and Dahmane, N. (2002a). Hedgehog–GLI signaling and the growth of the brain. *Nat. Rev. Neurosci.* 3, 24-33. 10.1038/nrn704 11823802

[DMM050323C73] Ruiz i Altaba, A., Sánchez, P. and Dahmane, N. (2002b). Gli and hedgehog in cancer: tumours, embryos and stem cells. *Nat. Rev. Cancer* 2, 361-372. 10.1038/nrc796 12044012

[DMM050323C74] Skowron, P., Farooq, H., Cavalli, F. M. G., Morrissy, A. S., Ly, M., Hendrikse, L. D., Wang, E. Y., Djambazian, H., Zhu, H., Mungall, K. L. et al. (2021). The transcriptional landscape of Shh medulloblastoma. *Nat. Commun.* 12, 1749. 10.1038/s41467-021-21883-0 33741928 PMC7979819

[DMM050323C75] Smoll, N. R. and Drummond, K. J. (2012). The incidence of medulloblastomas and primitive neurectodermal tumours in adults and children. *J. Clin. Neurosci.* 19, 1541-1544. 10.1016/j.jocn.2012.04.009 22981874

[DMM050323C76] Stemmer, M., Thumberger, T., del Sol Keyer, M., Wittbrodt, J. and Mateo, J. L. (2015). CCTop: An intuitive, flexible and reliable CRISPR/Cas9 target prediction tool. *PLoS ONE* 10, e0124633. 10.1371/journal.pone.0124633 25909470 PMC4409221

[DMM050323C77] Stoykova, A. and Gruss, P. (1994). Roles of Pax-genes in developing and adult brain as suggested by expression patterns. *J. Neurosci.* 14, 1395-1412. 10.1523/JNEUROSCI.14-03-01395.1994 8126546 PMC6577564

[DMM050323C78] Su, S., Chhabra, G., Singh, C. K., Ndiaye, M. A. and Ahmad, N. (2022). PLK1 inhibition-based combination therapies for cancer management. *Transl. Oncol.* 16, 101332. 10.1016/j.tranon.2021.101332 34973570 PMC8728518

[DMM050323C79] Susanto, E., Marin Navarro, A., Zhou, L., Sundström, A., van Bree, N., Stantic, M., Moslem, M., Tailor, J., Rietdijk, J., Zubillaga, V. et al. (2020). Modeling SHH-driven medulloblastoma with patient iPS cell-derived neural stem cells. *Proc. Natl. Acad. Sci. USA* 117, 20127-20138. 10.1073/pnas.1920521117 32747535 PMC7443968

[DMM050323C80] Taipale, J., Chen, J. K., Cooper, M. K., Wang, B., Mann, R. K., Milenkovic, L., Scott, M. P. and Beachy, P. A. (2000). Effects of oncogenic mutations in Smoothened and Patched can be reversed by cyclopamine. *Nature* 406, 1005-1009. 10.1038/35023008 10984056

[DMM050323C81] Tamayo-Orrego, L., Wu, C.-L., Bouchard, N., Khedher, A., Swikert, S. M., Remke, M., Skowron, P., Taylor, M. D. and Charron, F. (2016). Evasion of cell senescence leads to medulloblastoma progression. *Cell Rep.* 14, 2925-2937. 10.1016/j.celrep.2016.02.061 26997276

[DMM050323C82] Tamayo-Orrego, L., Gallo, D., Racicot, F., Bemmo, A., Mohan, S., Ho, B., Salameh, S., Hoang, T., Jackson, A. P., Brown, G. W. et al. (2020). Sonic hedgehog accelerates DNA replication to cause replication stress promoting cancer initiation in medulloblastoma. *Nat. Cancer* 1, 840-854. 10.1038/s43018-020-0094-7 35122047

[DMM050323C83] Ulloa, F. and Martí, E. (2010). Wnt won the war: antagonistic role of Wnt over Shh controls dorso-ventral patterning of the vertebrate neural tube. *Dev. Dyn.* 239, 69-76. 10.1002/dvdy.22058 19681160

[DMM050323C84] van Essen, M., Riepsaame, J. and Jacob, J. (2021). CRISPR-Cas gene perturbation and editing in human induced pluripotent stem cells. *CRISPR J.* 4, 634-655. 10.1089/crispr.2021.0063 34582693

[DMM050323C85] van Essen, M. J., Nayler, S., Apsley, E. J., Jacob, J. and Becker, E. B. E. (2022). Cerebellar modelling using human induced pluripotent stem cells. In *Measuring Cerebellar function (Neuromethods)* (ed. R. V. Sillitoe), pp. 1-21. Humana Press Inc.: New York. 10.1007/978-1-0716-2026-7_1

[DMM050323C86] Vaughan-Jackson, A., Stodolak, S., Ebrahimi, K. H., Browne, C., Reardon, P. K., Pires, E., Gilbert-Jaramillo, J., Cowley, S. A. and James, W. S. (2021). Differentiation of human induced pluripotent stem cells to authentic macrophages using a defined, serum-free, open-source medium. *Stem Cell Rep.* 16, 1735-1748. 10.1016/j.stemcr.2021.05.018 PMC8282471 34171284

[DMM050323C87] Vernay, B., Koch, M., Vaccarino, F., Briscoe, J., Simeone, A., Kageyama, R. and Ang, S.-L. (2005). Otx2 regulates subtype specification and neurogenesis in the midbrain. *J. Neurosci.* 25, 4856-4867. 10.1523/JNEUROSCI.5158-04.2005 15888661 PMC6724764

[DMM050323C88] Vladoiu, M. C., El-Hamamy, I., Donovan, L. K., Farooq, H., Holgado, B. L., Sundaravadanam, Y., Ramaswamy, V., Hendrikse, L. D., Kumar, S., Mack, S. C. et al. (2019). Childhood cerebellar tumours mirror conserved fetal transcriptional programs. *Nature* 572, 67-73. 10.1038/s41586-019-1158-7 31043743 PMC6675628

[DMM050323C89] Watson, L. M., Wong, M. M. K., Vowles, J., Cowley, S. A. and Becker, E. B. E. (2018). A simplified method for generating Purkinje cells from human-induced pluripotent stem cells. *Cerebellum* 17, 419-427. 10.1007/s12311-017-0913-2 29397531 PMC6028833

[DMM050323C90] Wechsler-Reya, R. J. and Scott, M. P. (1999). Control of neuronal precursor proliferation in the cerebellum by Sonic Hedgehog. *Neuron* 22, 103-114. 10.1016/S0896-6273(00)80682-0 10027293

[DMM050323C91] Yang, Z.-J., Ellis, T., Markant, S. L., Read, T.-A., Kessler, J. D., Bourboulas, M., Schüller, U., Machold, R., Fishell, G., Rowitch, D. H. et al. (2008). Medulloblastoma can be initiated by deletion of Patched in lineage-restricted progenitors or stem cells. *Cancer Cell* 14, 135-145. 10.1016/j.ccr.2008.07.003 18691548 PMC2538687

[DMM050323C92] Yeung, J., Ha, T. J., Swanson, D. J., Choi, K., Tong, Y. and Goldowitz, D. (2014). Wls provides a new compartmental view of the rhombic lip in mouse cerebellar development. *J. Neurosci.* 34, 12527-12537. 10.1523/JNEUROSCI.1330-14.2014 25209290 PMC4160781

[DMM050323C93] Zecevic, N. and Rakic, P. (1976). Differentiation of Purkinje cells and their relationship to other components of developing cerebellar cortex in man. *J. Comp. Neurol.* 167, 27-47. 10.1002/cne.901670103 818132

[DMM050323C94] Zhu, A., Ibrahim, J. G. and Love, M. I. (2019). Heavy-tailed prior distributions for sequence count data: removing the noise and preserving large differences. *Bioinformatics* 35, 2084-2092. 10.1093/bioinformatics/bty895 30395178 PMC6581436

