# Supplementary material for: PTCH1-mutant human cerebellar organoids exhibit altered neural development and recapitulate early medulloblastoma tumorigenesis
Source: Dis Model Mech. 2024 Feb 27;17(2):dmm050323. doi: 10.1242/dmm.050323 (PMC10924233; doi:10.1242/dmm.050323)
Supplement: Supplementary information [file dmm-17-050323-s1.pdf]

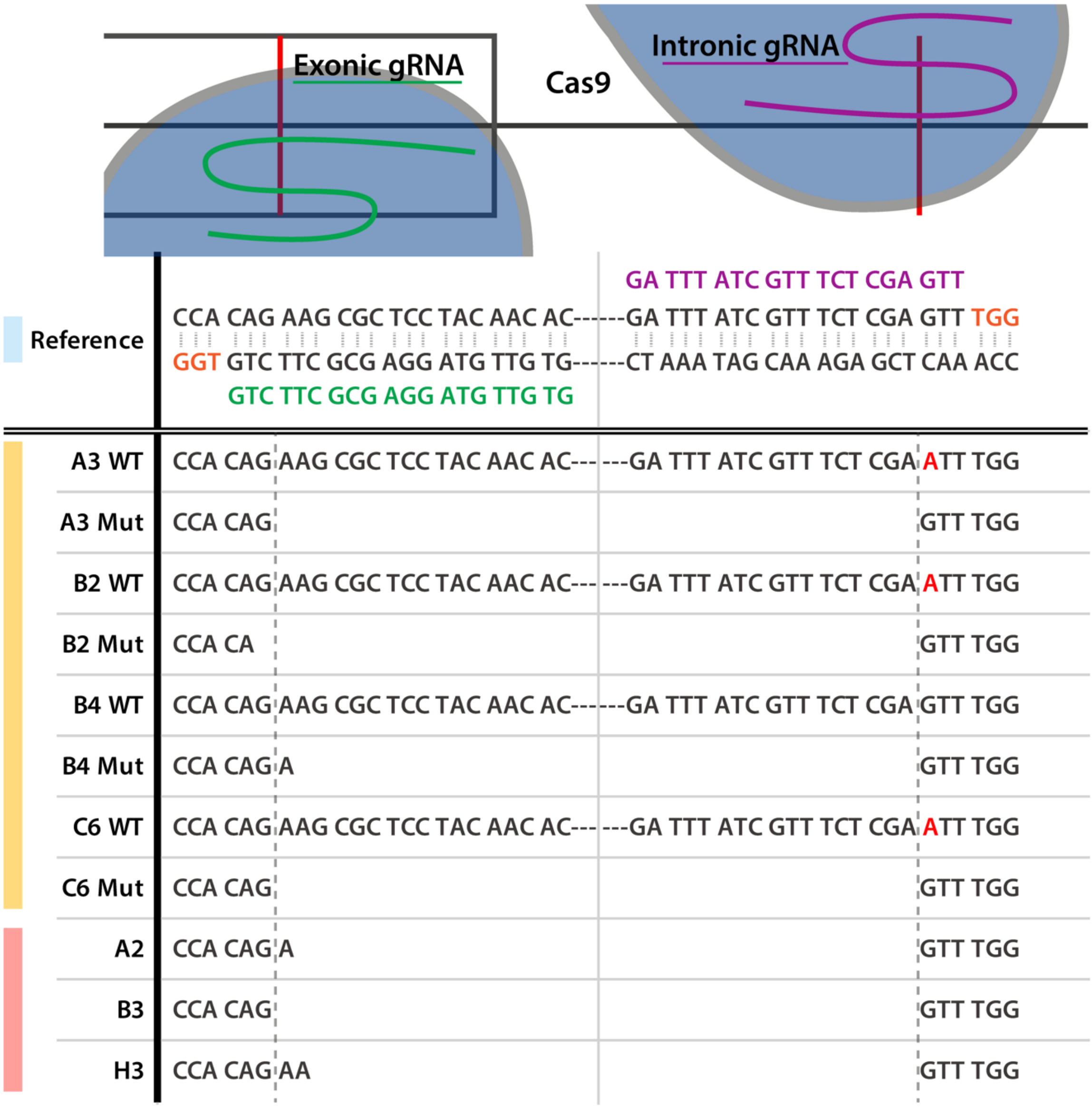

Fig. S1. Sanger sequencing of CRISPR target site in the *PTCH1* gene (related to Fig. 1) Top: Sequencing of control *PTCH1* sequence (reference). Guide RNA (gRNA) sequences (exonic in green, intronic in purple) and respective protospacer adjacent motifs (marked orange in reference sequence) are displayed with the coding strand of the reference sequence on top. Below: Sequencing of mutant clones. The expected cut sites are depicted as vertical dashed lines. Sanger sequencing revealed uniform cutting at the intronic target site compared to more heterogeneous cutting at the exonic target site. The genomic DNA region between the target sites is absent in the mutant amplicons (mut). Point mutations at the intronic target site resulting from the Cas9-induced double strand break are detected in heterozygous clones (G>A) (marked in red).

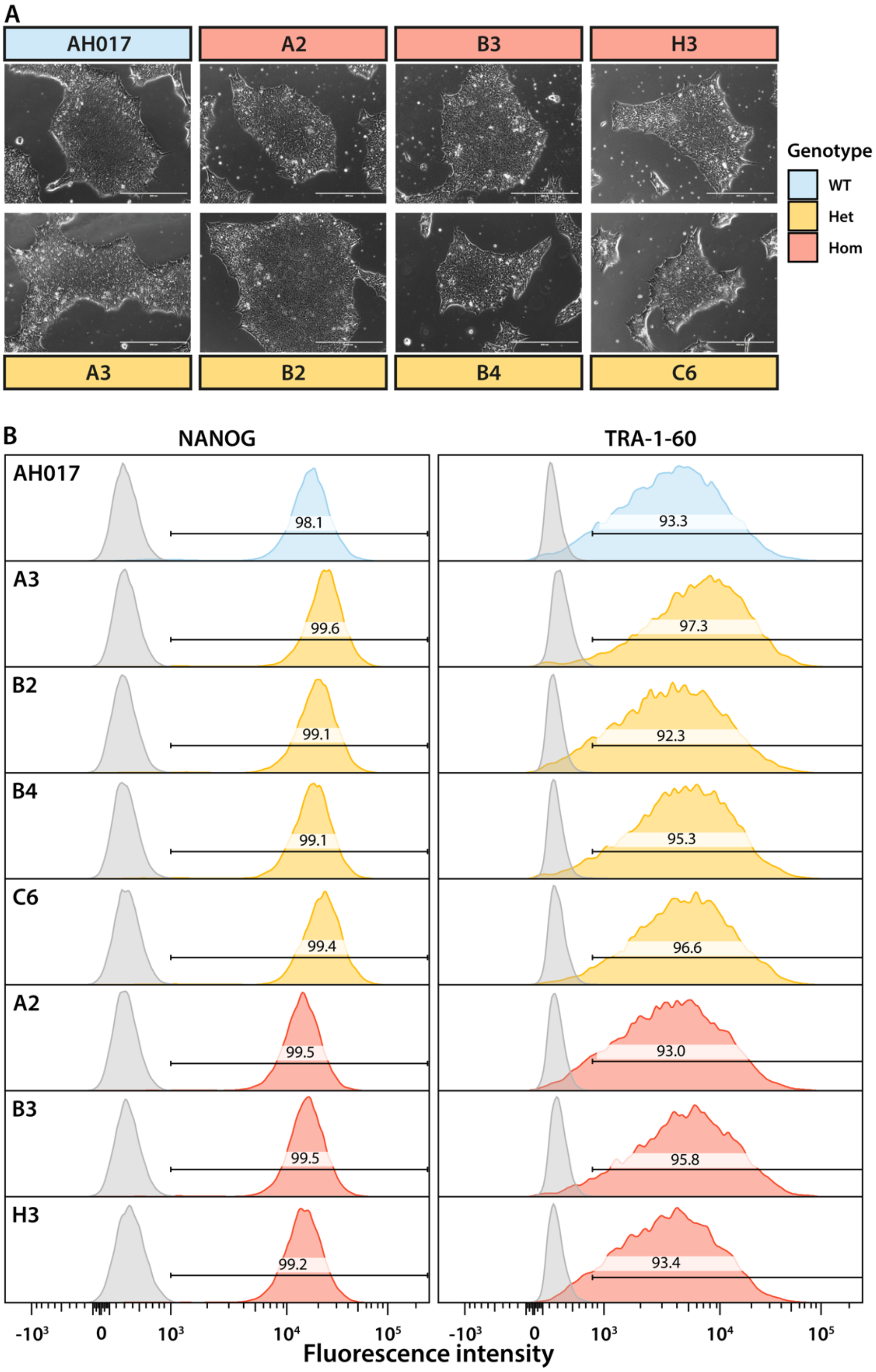

Fig. S2. PTCH1 mutant clones maintain iPSC morphology and pluripotency marker expression (related to Fig. 1)

A) Brightfield images of iPSC clones. Scale bars 1000µm. All clones display normal iPSC morphology with large nucleus-to-cytoplasm ratio and growth in densely packed colonies.

B) A total of 50,000 cells were measured by flow cytometry. Density plot shows the distribution of fluorescence intensity along the x-axis. Coloured histograms represent cells stained with antibodies targeting NANOG (left) or TRA-1-60 (right). Grey histograms represent cells stained with isotype control antibodies to assess non-specific binding and autofluorescence. The percentage of cells with fluorescence intensities above the isotype control condition are depicted.

Disease Models & Mechanisms • Supplementary information

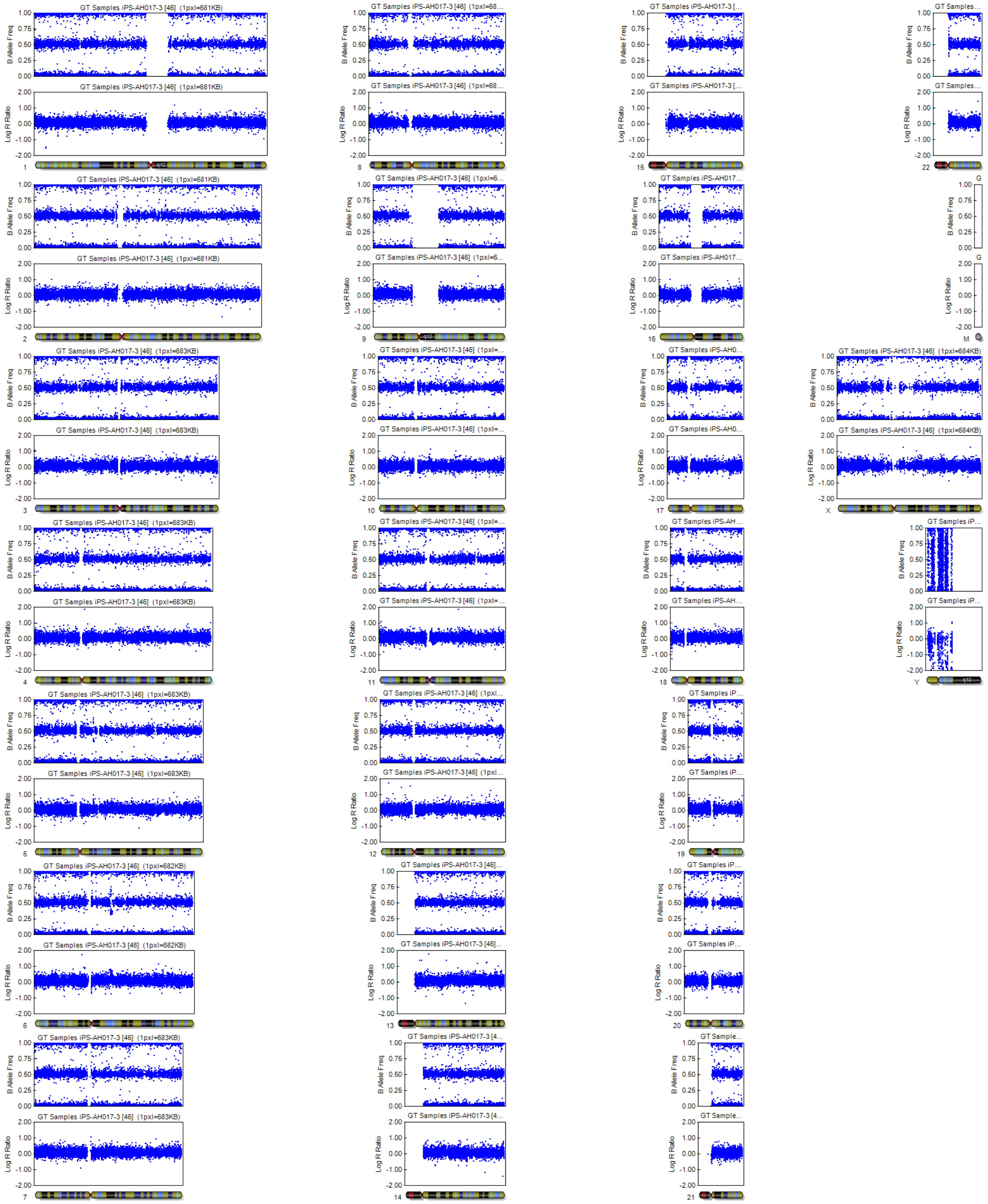

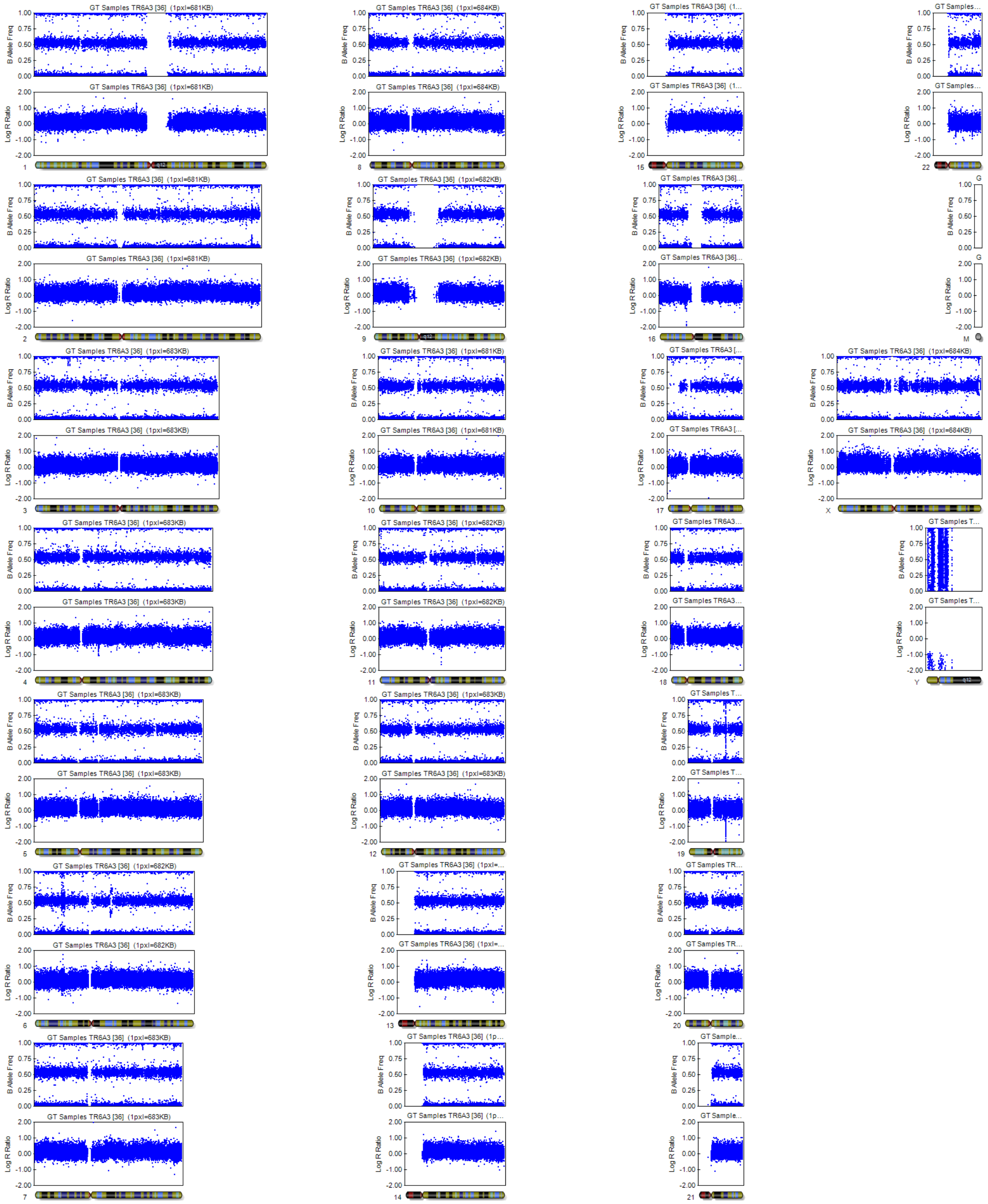
GT Samples TR6A3 [36] (1pxl=681KB)
B Allele Freq
Log R Ratio
1.00
0.75
0.50
0.25
0.00
2.00
1.00
0.00
-1.00
-2.00
1
2
3
4
5
6
7
8
9
10
11
12
13
14
15
16
17
18
19
20
21
22
X
Y
M
G

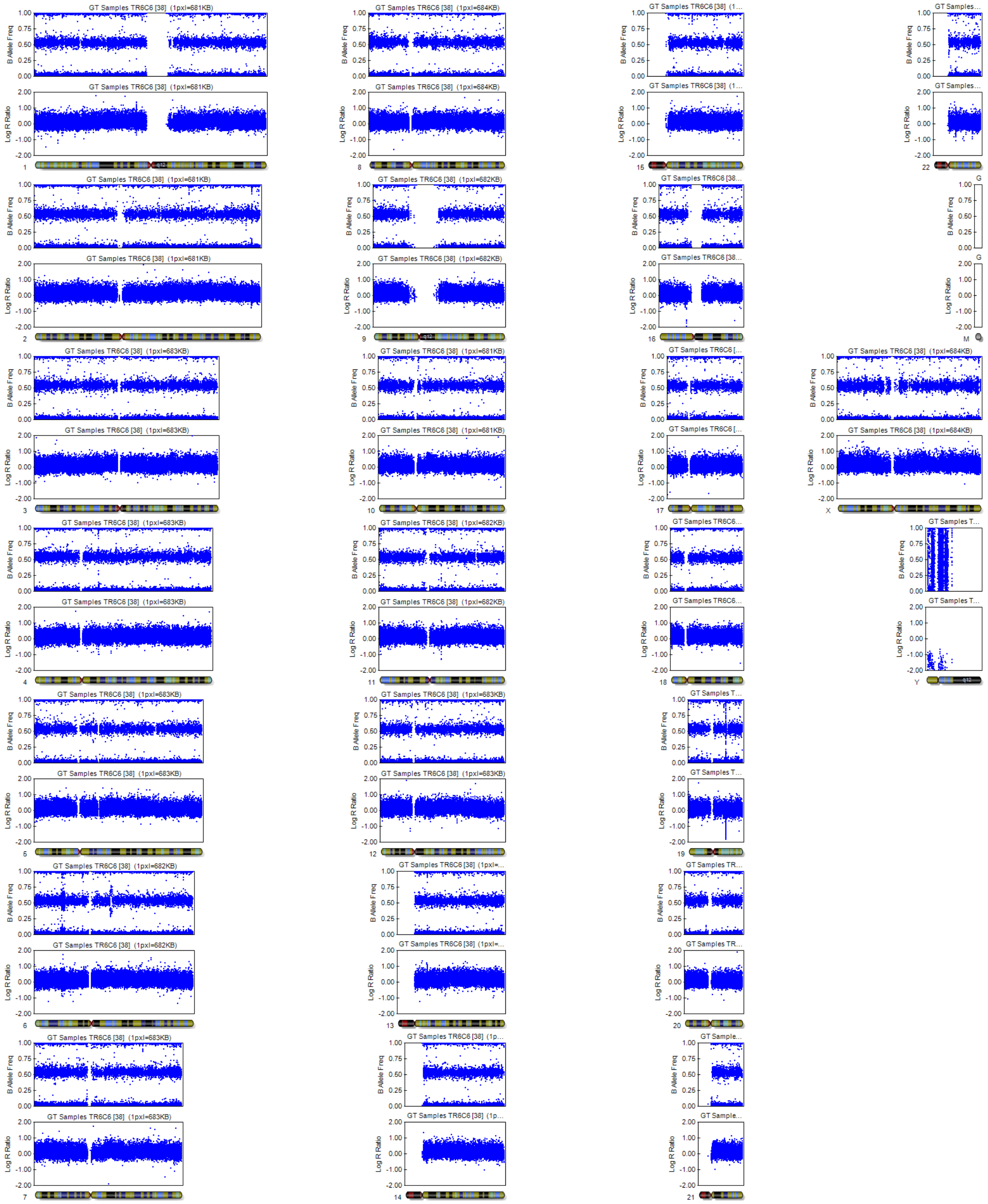

Disease Models & Mechanisms • Supplementary information

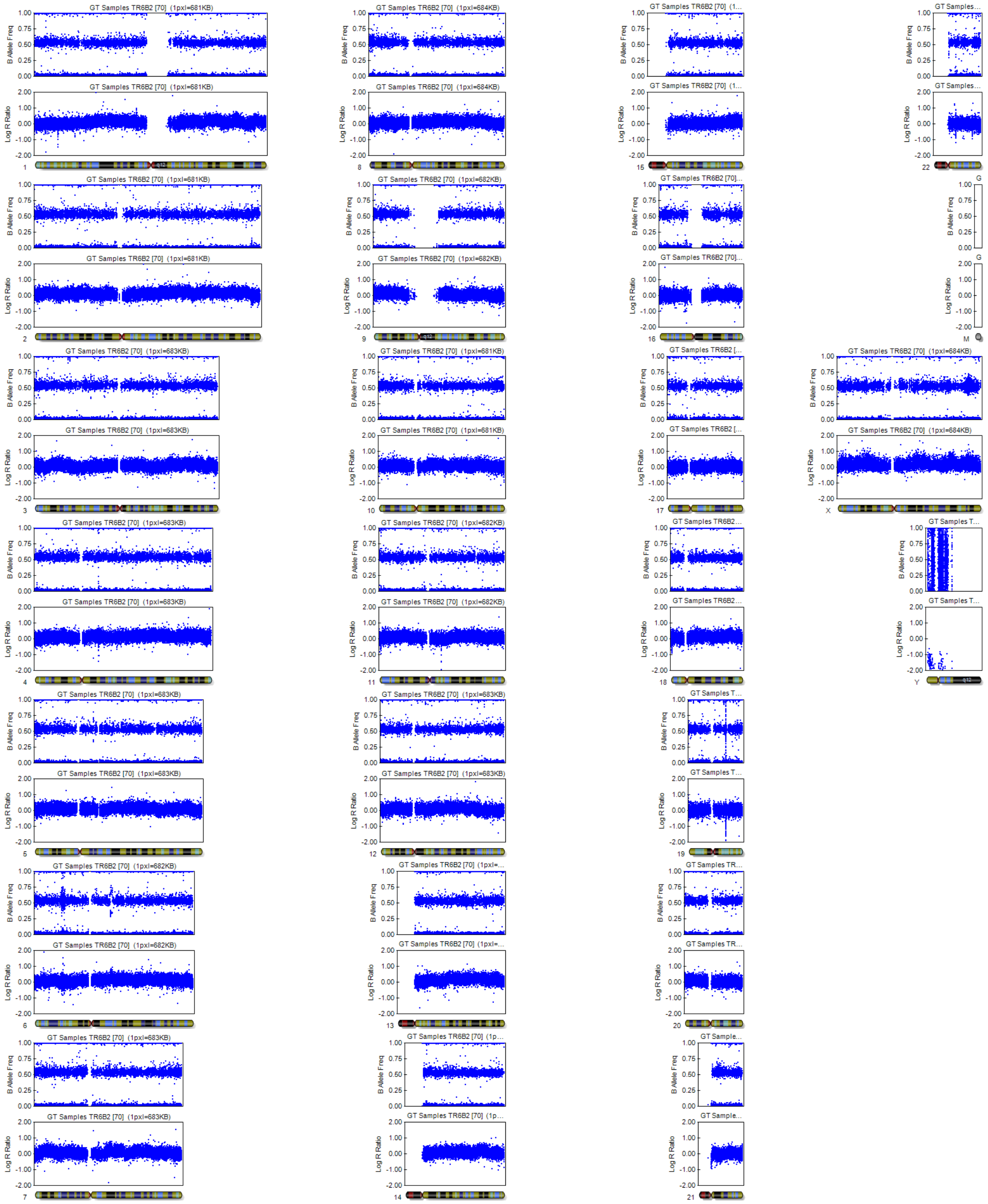
GT Samples TR6B2 [70] (1pxl=681KB)
B Allele Freq
Log R Ratio

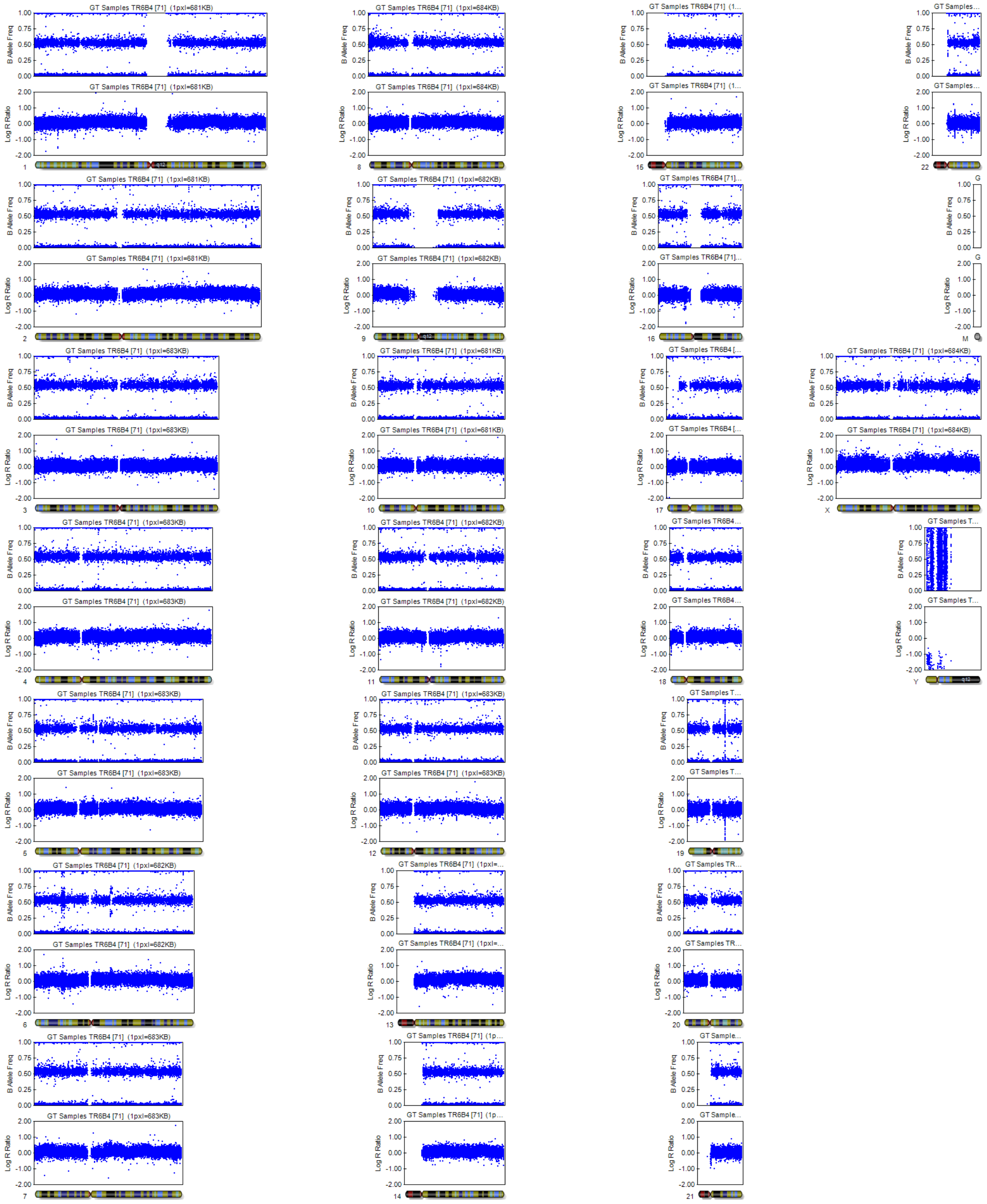

Disease Models & Mechanisms • Supplementary information

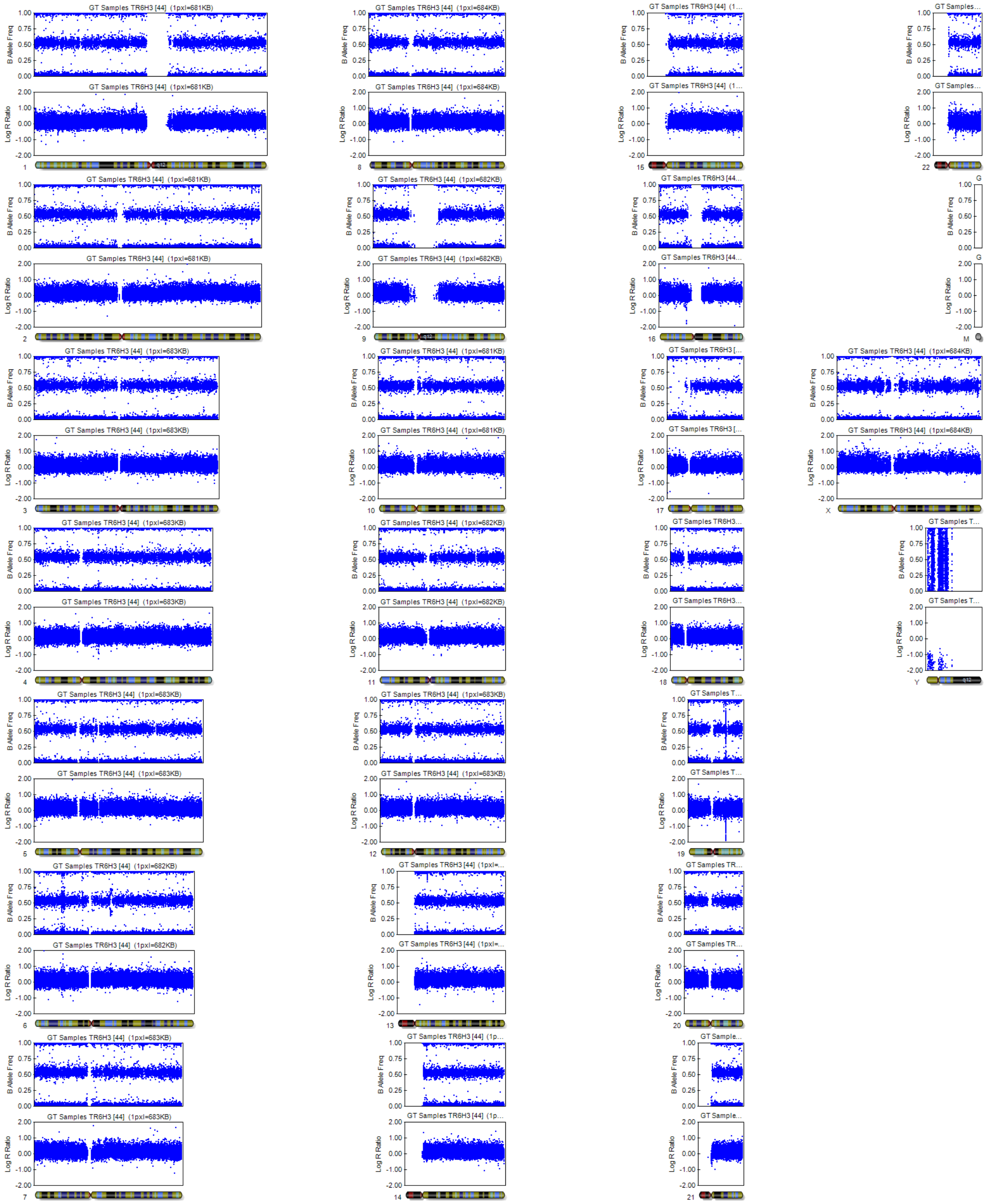

Disease Models & Mechanisms • Supplementary information

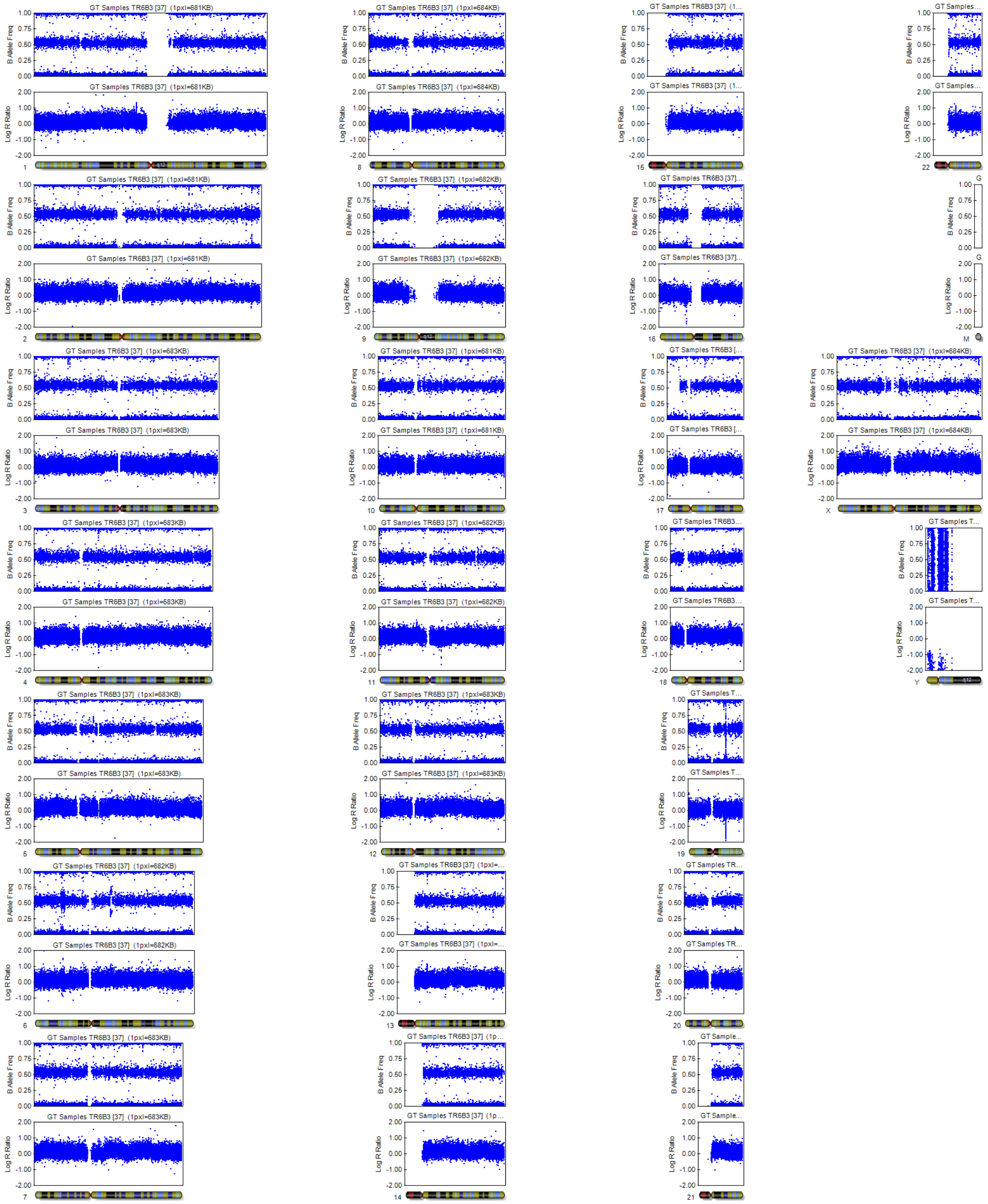
GT Samples TR6B3 [37] (1pxl=681KB)
B Allele Freq
Log R Ratio
1 2 3 4 5 6 7 8 9 10 11 12 13 14 15 16 17 18 19 20 21 22 X Y M

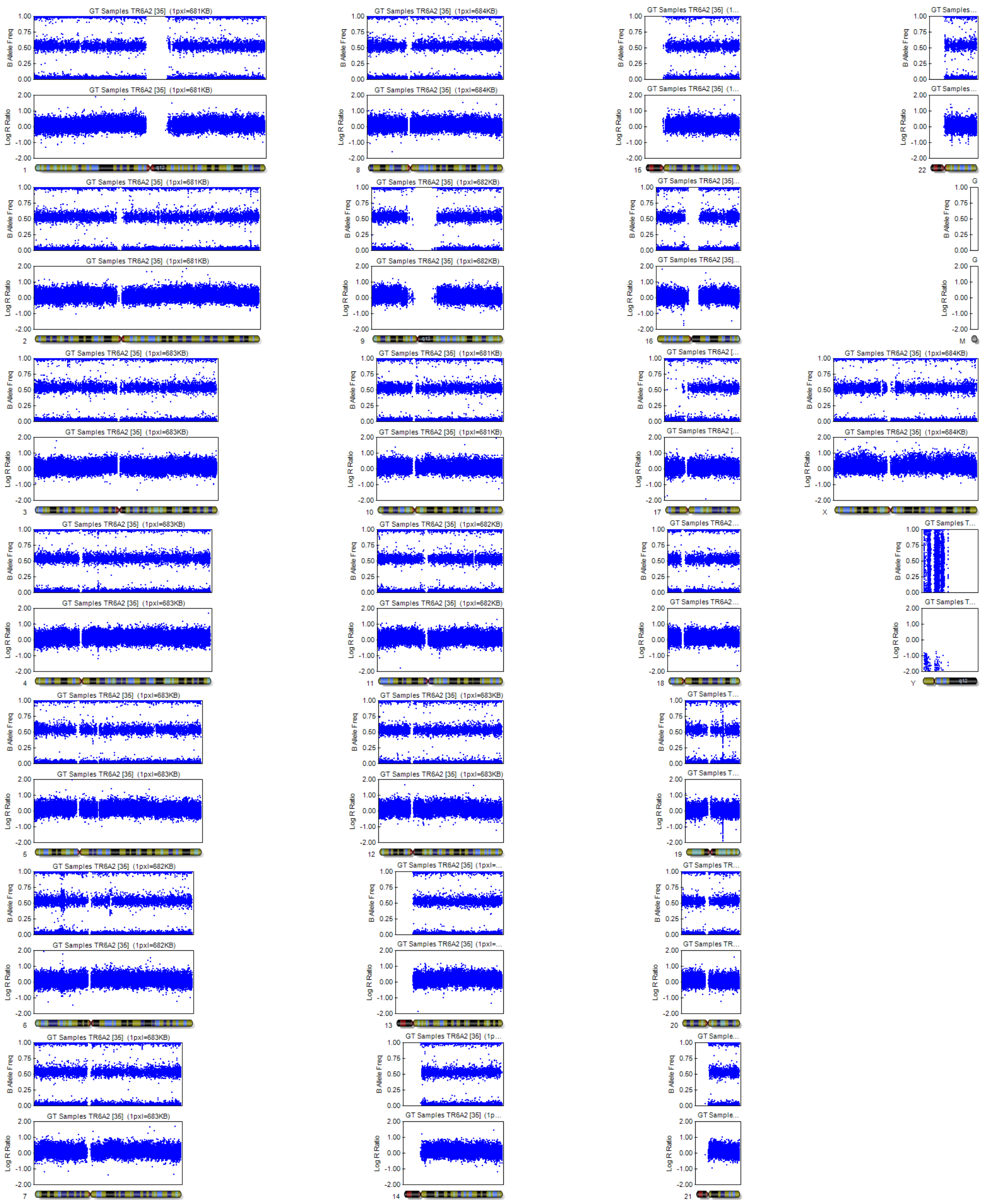

Fig. S3. SNP array to test for chromosomal aberrations in gene edited iPSC clones (related to Fig. 1)

For each chromosome, the first dot plot displays the B-allele frequency and indicates whether the SNV is heterozygous (data points fall at around 0.5) or homozygous (data point at around 0.0 of 1.0). The second plot displays the log R value (first dot plot, indicated with R on the left side) is given, representing the probe intensity of individual SNVs. Chromosomal gains are identified by doubling of the log R value while halving the R value indicates loss. The different clones are given in the following order: control line: AH017-3; heterozygous (*PTCH1*$^{+/-}$) clones: A3, C6, B2, B4; homozygous (*PTCH1*$^{-/-}$) clones: H3, B3, A2.

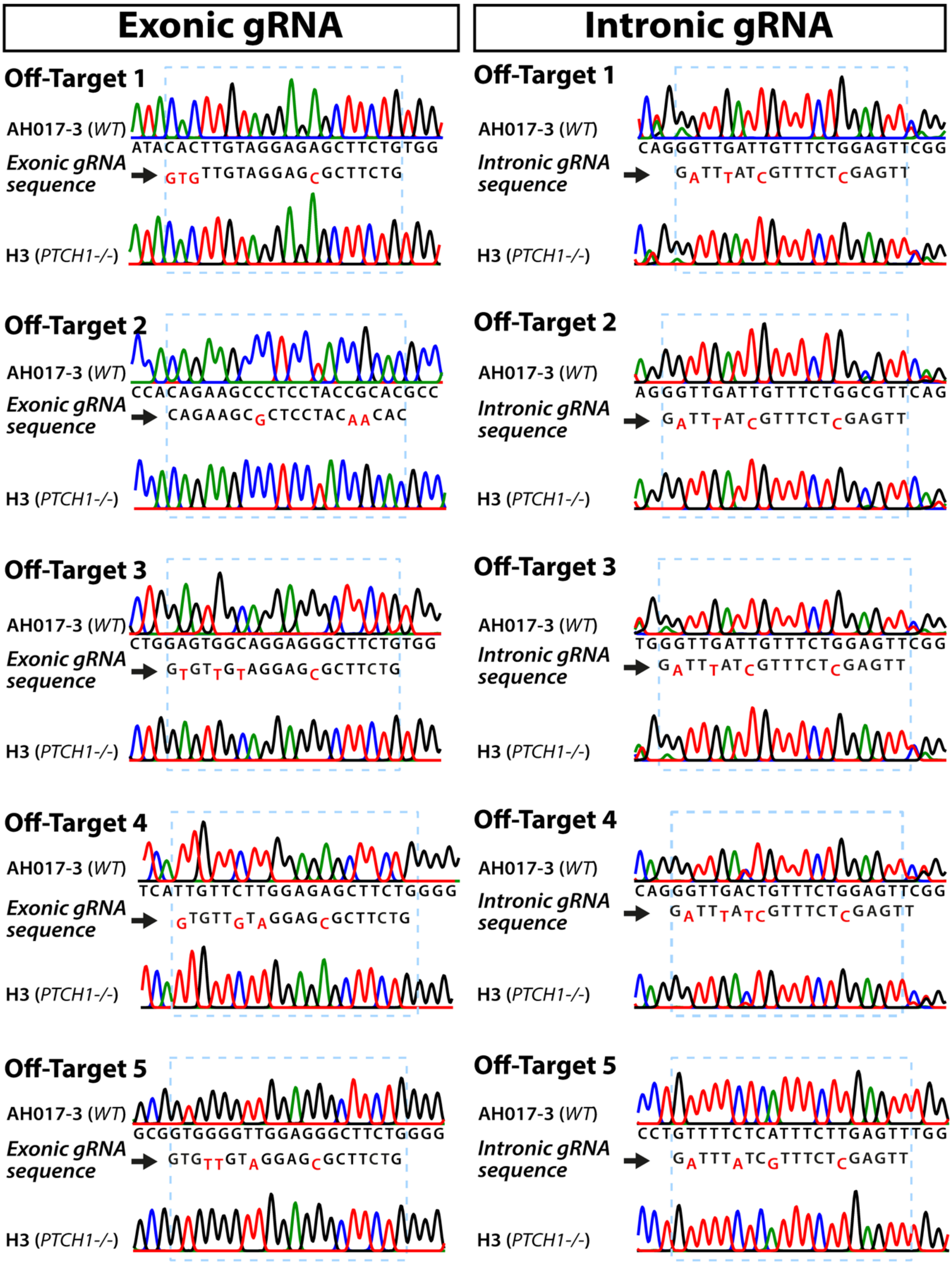

Fig. S4. Sanger sequencing does not reveal off-target CRISPR-Cas activity (related to Fig. 1)

Sanger sequencing of the top five off-target sites of each guide RNA. The alignment with each respective gRNA is shown below the wildtype (WT) strand with mismatches shown in red. Sequence traces of both the AH017-3 WT and tested homozygous mutant (H3) were identical.

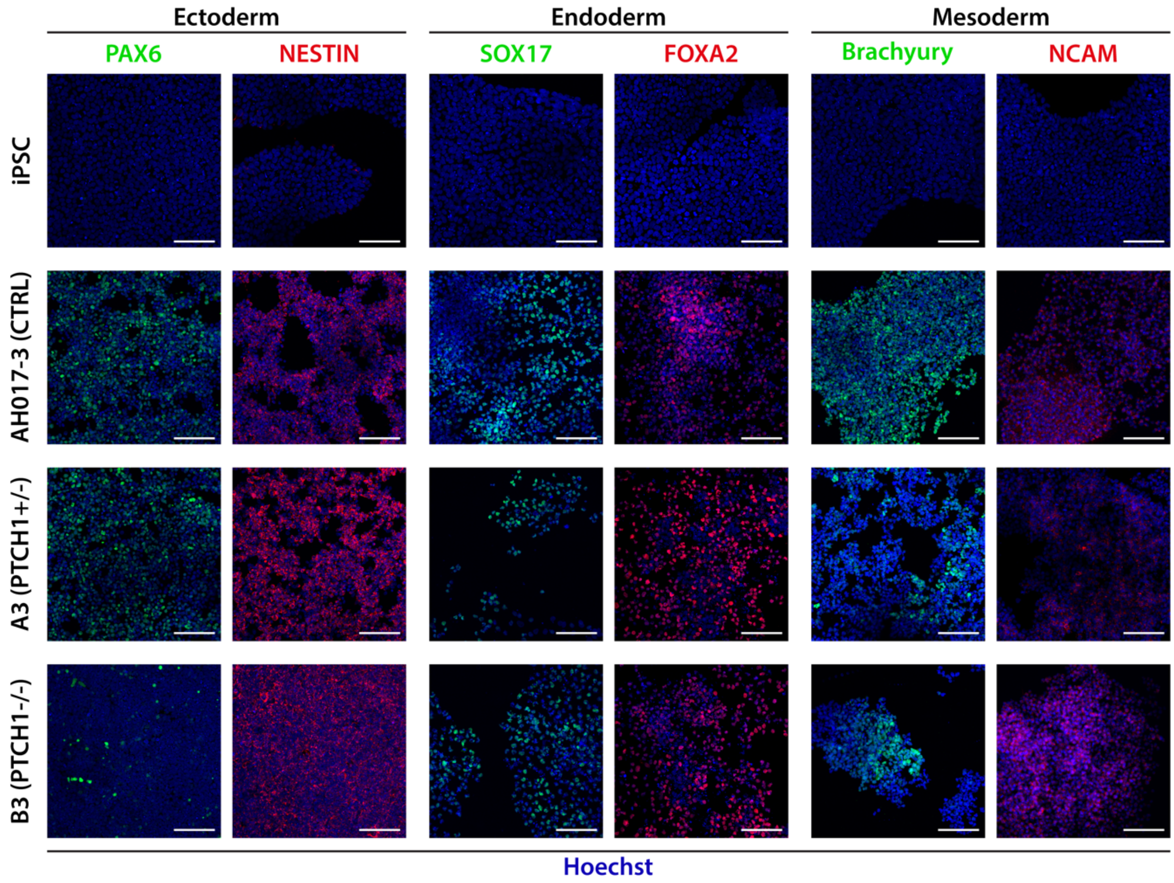

Fig. S5. Differentiation of iPSC clones into the three germ layers shows maintained pluripotent capacity (related to Fig. 1)

Immunostaining of iPSCs differentiated into either ectodermal, endodermal or mesodermal stem cells using the STEMdiff™ Trilineage Differentiation Kit. Cells are immunostained using antibodies specific to ectodermal progenitors (PAX6: green, NESTIN: red), endodermal progenitors (SOX17: green, FOXA2: red) or mesodermal progenitors (Brachyury: green, NCAM: red). Nuclei are visualised in blue by Hoechst staining. Scale bars 150µm.

Disease Models & Mechanisms • Supplementary information

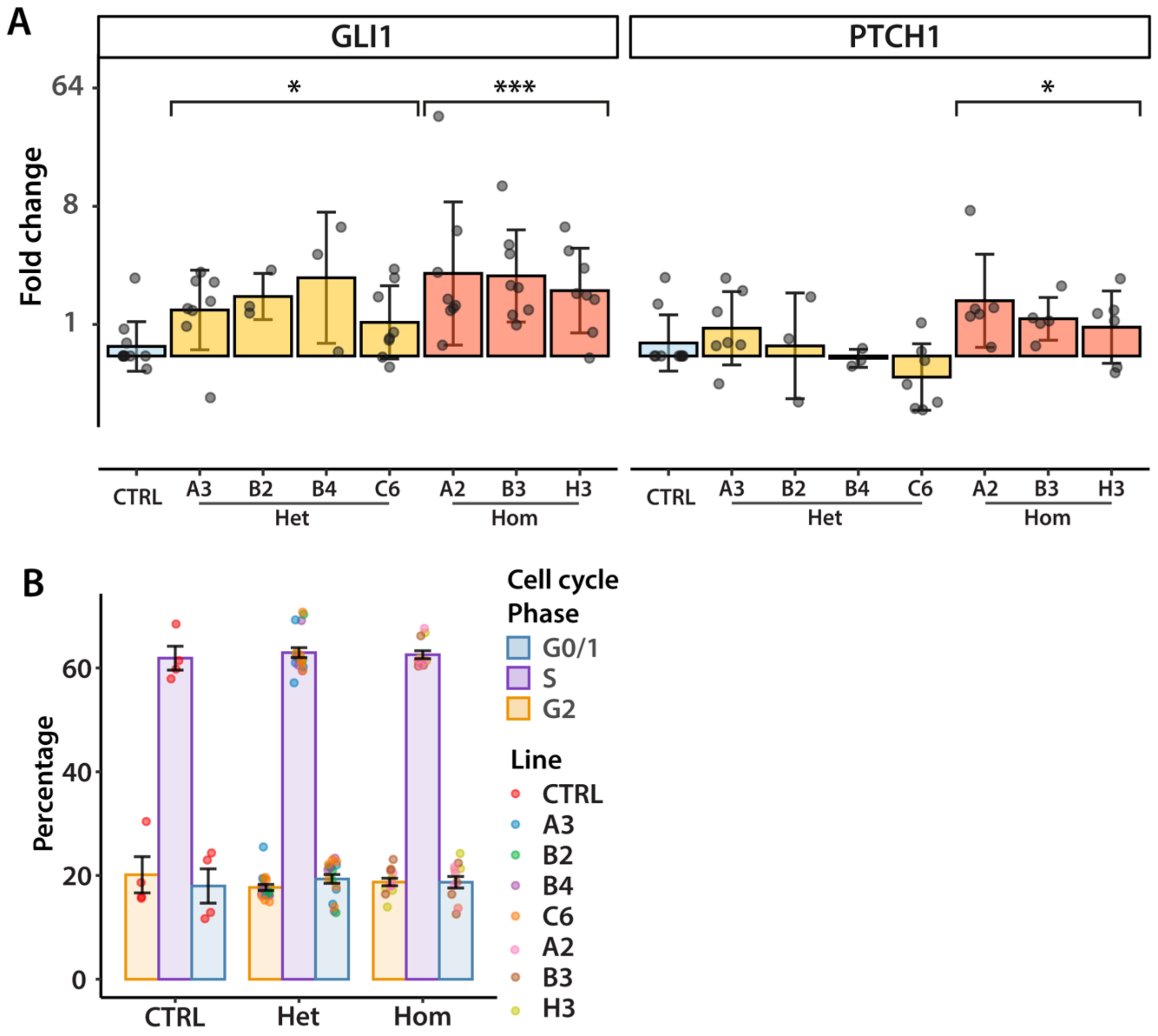

Fig. S6. Modest upregulation of SHH signalling in *PTCH1*-mutant iPSC with maintained cell cycle profile (related to Fig. 1)

(A) Messenger RNA expression of *GLI1* and *PTCH1* as measured by RT-qPCR. Expression is relative to the parent line (CTRL) and normalized using *ACTB* and *GAPDH*. Data points are generated from independent passages. n>3 for all clones. Statistically significant differences on the genotype level are calculated on the ΔΔCt values by one way ANOVA: *GLI1* (F(2,42)=11.821, p<0.001), Dunnett's post-hoc test (CTRL vs Het: p=0.014, 95% CI [-2.0990, -0.2172]; CTRL vs Hom: p<0.001, 95% CI [-2.9498, -1.0680]); *PTCH1* (F(2,32)=7.685, p=0.002), Dunnett's post-hoc test (CTRL vs Hom: p=0.01, 95% CI [--1.87930, -0.23886]). Statistical significance of Dunnett's post-hoc test is indicated as *p<0.05, ***p<0.001. Error bars represent standard deviation.

(B) Proportions of cells in G0/1 phase (orange), S phase (purple) and G2 phase (blue) as percentage of whole. Data points are generated from independent passages. n=3 for all clones. Error bars indicate standard error of the mean.

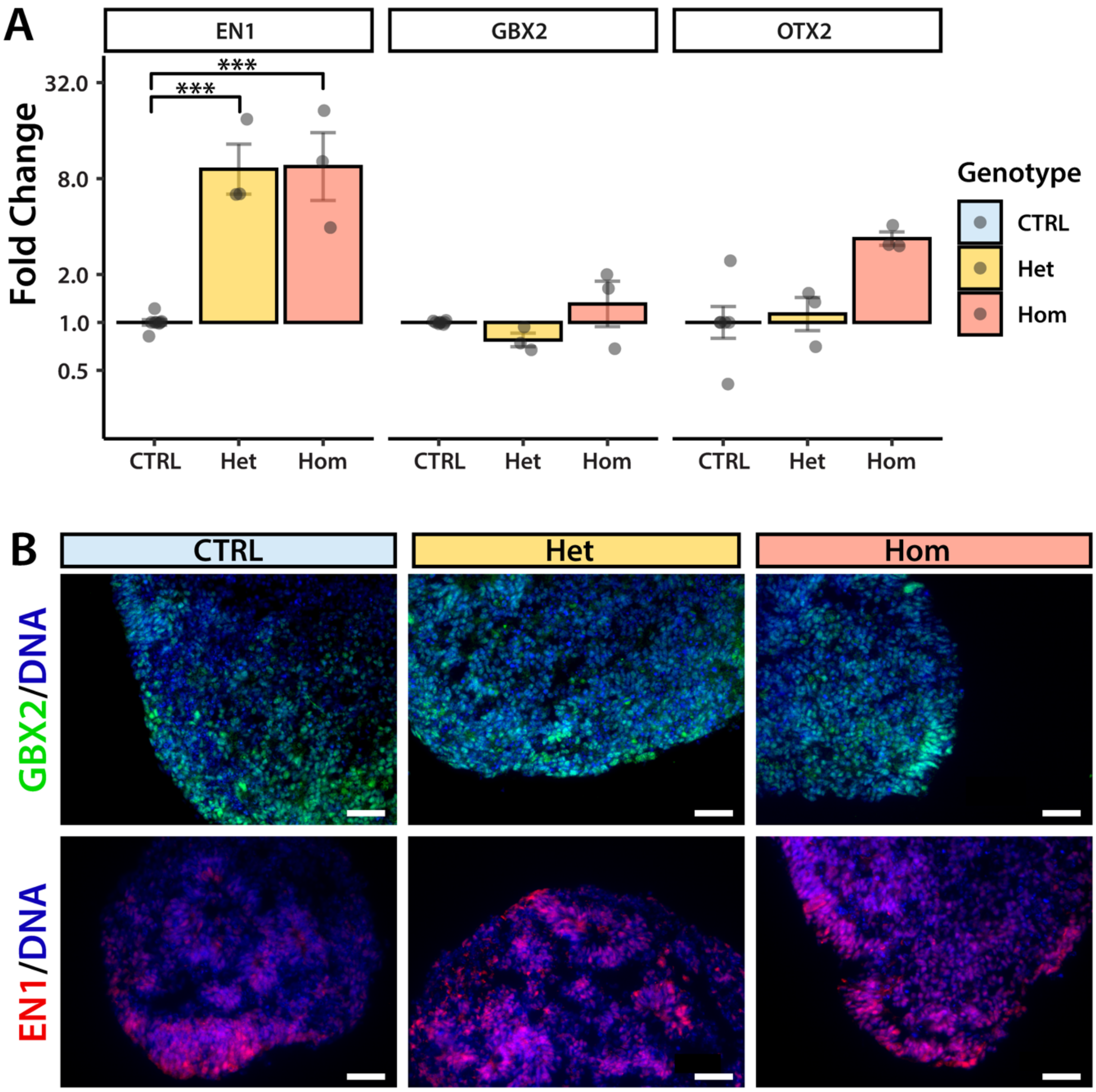

Fig. S7. Midbrain-hindbrain markers are expressed by *PTCH1* mutant organoids (related to Fig. 2)

(A) Expression of *EN1, GBX2* and *OTX2* at day 21 relative to iPSC in heterozygous (Het) and homozygous (Hom) organoids as measured by RT-qPCR and normalised to *GAPDH* and *ACTB*. Data are composed of three or more biological replicates from separate differentiations. Statistical significance was computed by one-way ANOVA: EN1 ($F(2,8)=23.676$, $p<0.001$), Dunnett's post-hoc test (CTRL vs Het: $p<0.001$, 95% CI [-4.7184, -1.6742]; CTRL vs Hom: $p<0.001$, 95% CI -4.7728, -1.7287]). Statistical significance of Dunnett's post-hoc test is indicated as ***$p<0.001$. Error bars indicate standard deviation.

(B) Immunofluorescence staining of day 21 organoids with antibodies specific to the hindbrain markers GBX2 (green) and EN1 (red). Nuclei are visualised in blue by Hoechst staining. Scale bars 150μm.

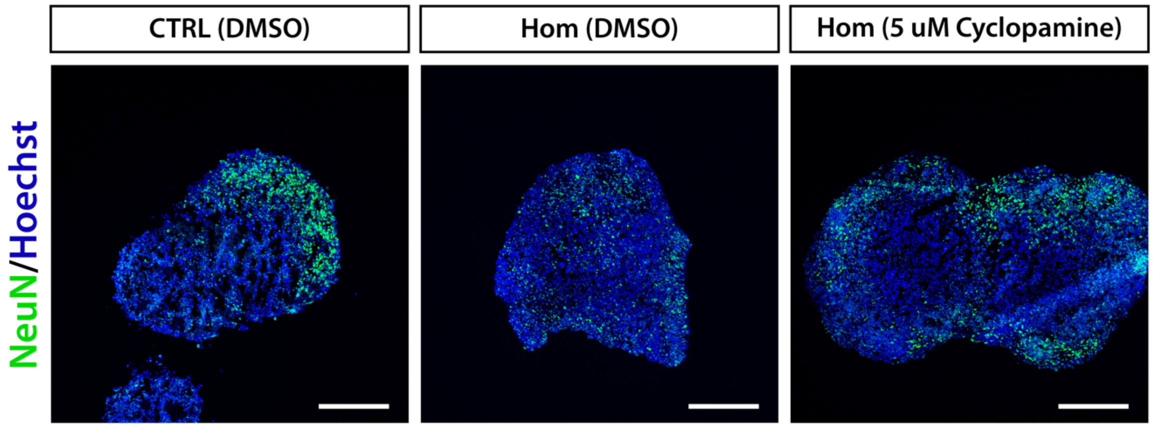

Fig. S8. Neuronal marker expression is maintained in Cyclopamine-treated *PTCH1*-/- organoids (related to Fig. 4)

Immunofluorescence staining of day 35 organoids with antibodies specific to the pan-neuronal marker NeuN (green). Nuclei are visualised in blue by Hoechst staining. Scale bars 150µm.

Disease Models & Mechanisms • Supplementary information

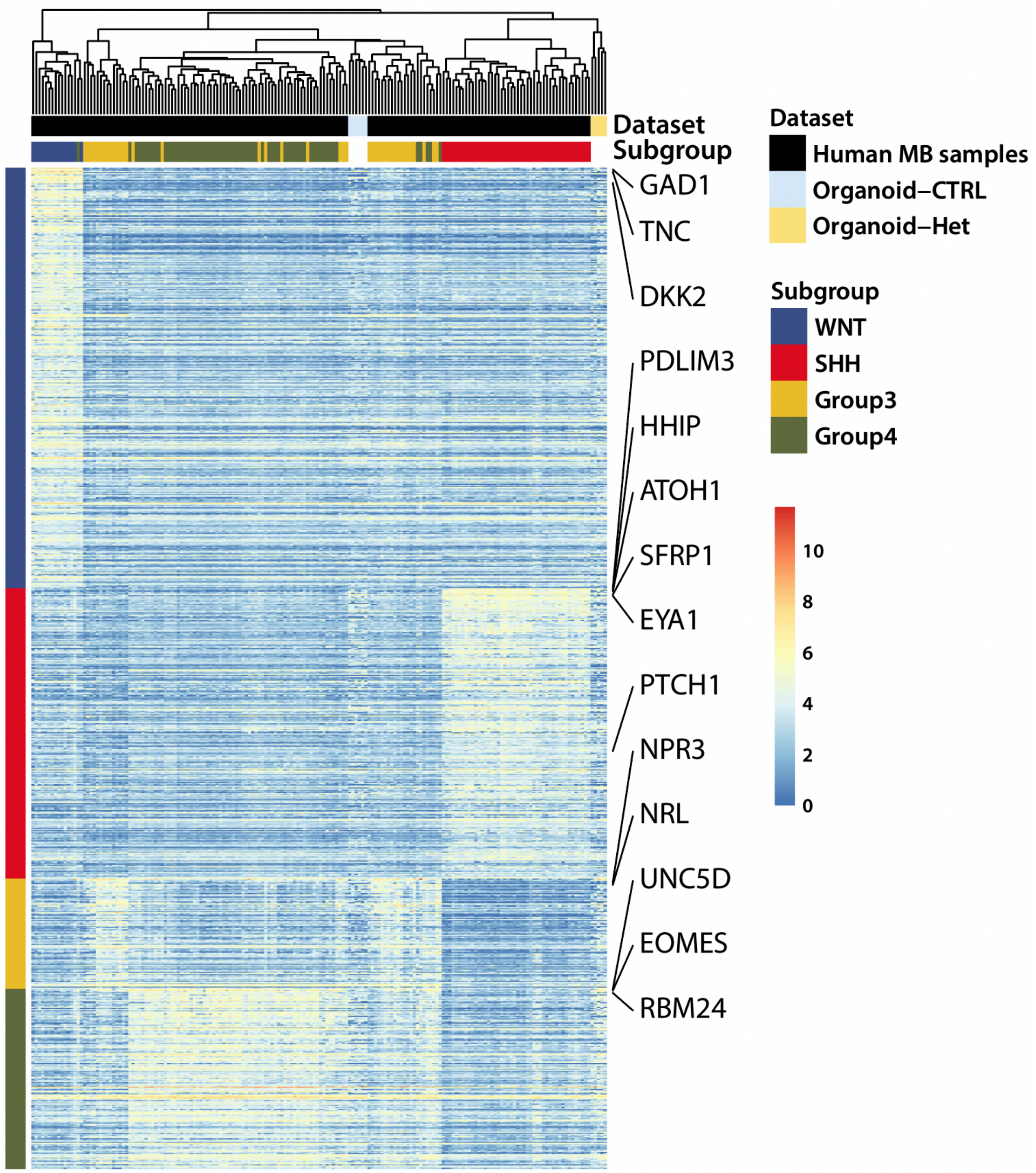

Fig. S9. *PTCH1* heterozygous organoids cluster with human SHH-MB samples (related to Fig. 6)

CTRL and *PTCH1+/-* heterozygous organoids were clustered with human medulloblastoma (MB) RNA-sequencing samples using an unsupervised hierarchical clustering method based on the normalized gene expression of 917 subgroup specific MB gene markers. The top subtype-specific markers are indicated on the right of the heatmap.

Table S1. Differentially expressed genes in homozygous clones

Available for download at

https://journals.biologists.com/dmm/article-lookup/doi/10.1242/dmm.050323#supplementary-data

Table S2. Differentially expressed genes in heterozygous clones

Available for download at
https://journals.biologists.com/dmm/article-lookup/doi/10.1242/dmm.050323#supplementary-data

Table S3. Primers used in this study

Gene	Fw/Rv	Sequence	Type
ACTB	Fw	GCCGGGACCTGACTGACTAC	RT-qPCR
ACTB	Rv	TTCTCCTTAATGTCACGCACGAT	RT-qPCR
ATOH1	Fw	TGTTATCCCGTCGTTCAACAAC	RT-qPCR
ATOH1	Rv	TGGGCGTTTGTAGCAGCTC	RT-qPCR
BARHL1	Fw	TTCTGATCAGGGACATCCTTGCC	RT-qPCR
BARHL1	Rv	TGCCACTTTTGTCCAGCTTGTC	RT-qPCR
EN1	Fw	CGTGGTCAAAACTGACTCGC	RT-qPCR
EN1	Rv	CGCTTGTCCTCCTTCTCGTT	RT-qPCR
GAPDH	Fw	GGAAGGTGAAGGTCGGAGTC	RT-qPCR
GAPDH	Rv	GTTGAGGTCAATGAAGGGGTC	RT-qPCR
GBX2	Fw	AAAGAGGGCTCGCTGCTC	RT-qPCR
GBX2	Rv	GGTCGTCTTCCACCTTTGAC	RT-qPCR
GLI1	Fw	CCCAGTACATGCTGGTGGTT	RT-qPCR
GLI1	Rv	GCTTTACTGCAGCCCTCGT	RT-qPCR
KIRREL2	Fw	CCTGAAGAAGAGGAGACAGGC	RT-qPCR
KIRREL2	Rv	TCCTCCAGAACCAGATCACTG	RT-qPCR
NKX2.1	Fw	CAAAGGCCAAACTGCTGGAC	RT-qPCR
NKX2.1	Rv	TGAGATTGGATGCGCTTGGT	RT-qPCR
NKX2.2	Fw	AGCTTCGCTTCTTTGCCTCT	RT-qPCR
NKX2.2	Rv	GGGGTCGGTCTTTTTCTCGT	RT-qPCR
OTX2	Fw	CACTTCGGGTATGGACTTGC	RT-qPCR
OTX2	Rv	GTGAACGTCGTCCTCTCCC	RT-qPCR
PAX6	Fw	AGCTAGCTCACAGCGGGG	RT-qPCR
PAX6	Rv	TCTGATGGAGCCAGTCTCGT	RT-qPCR
PTCH1	Fw	TACTGCTCACACATCAGCCAG	Genotyping
PTCH1	Rv	AGTTCAGCAAGGGCAATTCAA	Genotyping
PTCH1	Fw	GGCAGCGGTAGTAGTGGTGTTC	RT-qPCR
PTCH1	Rv	TGTAGCGGGTATTGTCGTGTGTG	RT-qPCR
PTCH1	Fw	ACATGTACAACAGGCAGTGGA	Exon 3 expr.
PTCH1	Rv	ATTTCGCCCCTTCCCAGAAG	Exon 3 expr
PTCH1	Fw	TTTGCGGTGGACAAACTTCG	Total *PTCH1* expr.
PTCH1	Rv	TTCAGCATTTCCTCCCAGCT	Total *PTCH1* expr.
PTCH2	Fw	GATGGGGCCATCTCCACATT	RT-qPCR
PTCH2	Rv	CGCCGCAAAGAAGTACCTTACA	RT-qPCR
SHH	Fw	CTCGCTGCTGGTATGCTCG	RT-qPCR
SHH	Rv	ATCGCTCGGAGTTTCTGGAGA	RT-qPCR
SIX3	Fw	AGCAGAAGACGCATTGCTTC	RT-qPCR
SIX3	Rv	ACCAGTTGCCTACTTGTGTG	RT-qPCR

Disease Models & Mechanisms • Supplementary information

Table S4. Primary antibodies used

Target	Catalogue number	Manufacturer	Host species	Dilution
EN1	ab190080-200ul	Abcam	Rabbit	1:250
GBX2	H00002637-M01	Novus bio	Mouse	1:200
KIRREL2	AF2930	RnD systems	Goat	1:500
PAX6	GTX113241	Genetex	Rabbit	1:1,000
CCNB1	05-373	Milipore	Mouse	1:1,000
TUBB3	GT1338	Genetex	Mouse	1:1,000
NKX2-2	ab191077	Abcam	Rabbit	1:1,000
NeuN	Ab104224	Abcam	Rabbit	1:1,000
NESTIN	MAB5326	Sigma	Mouse	1:200
SOX17	AF1924-SP	RnD systems	Goat	1:100
FOXA2	AF2400	RnD systems	Goat	1:200
Brachyury	AF2085-SP	RnD systems	Goat	1:100
NCAM	ab75813	Abcam	Rabbit	1:200

Table S5. Secondary antibodies used

Target species	Host species	Conjugate	Catalogue number	Manufacturer	Dilution
Rabbit	Goat	Alexa Fluor 594	A11037	Invitrogen	1:1,000
Mouse	Goat	Alexa Fluor 488	A11029	Invitrogen	1:1,000
Goat	Donkey	Alexa Fluor 594	A11058	Invitrogen	1:1,000
Rabbit	Donkey	Alexa Fluor 488	A21206	Invitrogen	1:1,000
